# MHC genotyping of non-model organisms using next-generation sequencing: a new methodology to deal with artefacts and allelic dropout

**DOI:** 10.1186/1471-2164-14-542

**Published:** 2013-08-09

**Authors:** Simone Sommer, Alexandre Courtiol, Camila J Mazzoni

**Affiliations:** 1 Evolutionary Genetics, Leibniz-Institute for Zoo and Wildlife Research (IZW), Alfred-Kowalke-Straße 17, D-10315 Berlin, Germany; 2 Berlin Center for Genomics in Biodiversity Research, Koenigin-Luise-Straße 6-8, D-14195 Berlin, Germany

**Keywords:** Major histocompatibility complex, Next-generation sequencing, 454 pyrosequencing, Molecular cloning, PCR and sequencing artefacts, Amplification efficiency, Allelic dropout, Rodent, *Delomys sublineatus*

## Abstract

**Background:**

The Major Histocompatibility Complex (MHC) is the most important genetic marker to study patterns of adaptive genetic variation determining pathogen resistance and associated life history decisions. It is used in many different research fields ranging from human medical, molecular evolutionary to functional biodiversity studies. Correct assessment of the individual allelic diversity pattern and the underlying structural sequence variation is the basic requirement to address the functional importance of MHC variability. Next-generation sequencing (NGS) technologies are likely to replace traditional genotyping methods to a great extent in the near future but first empirical studies strongly indicate the need for a rigorous quality control pipeline. Strict approaches for data validation and allele calling to distinguish true alleles from artefacts are required.

**Results:**

We developed the analytical methodology and validated a data processing procedure which can be applied to any organism. It allows the separation of true alleles from artefacts and the evaluation of genotyping reliability, which in addition to artefacts considers for the first time the possibility of allelic dropout due to unbalanced amplification efficiencies across alleles. Finally, we developed a method to assess the confidence level per genotype a-posteriori, which helps to decide which alleles and individuals should be included in any further downstream analyses. The latter method could also be used for optimizing experiment designs in the future.

**Conclusions:**

Combining our workflow with the study of amplification efficiency offers the chance for researchers to evaluate enormous amounts of NGS-generated data in great detail, improving confidence over the downstream analyses and subsequent applications.

## Background

The Major Histocompatibility Complex (MHC) is a multigene family responsible for the adaptive immune response in vertebrate hosts [1] and has become the most preferred marker to study patterns of adaptive genetic variation related to health issues and life history decisions [2]. A hallmark of MHC genes is the high level of polymorphism observed in most natural populations caused by positive selection, gene duplication, recombination and gene conversion [3]. The variability in the MHC is represented by the number of alleles present both at the individual and population level, the excess of heterozygosity, the sequence divergence between alleles, as well as the number of locus duplications [1]. The number of MHC genes can differ greatly within and between species, especially in the classical MHC genes, such as class I and class II loci, probably due to their functional importance in pathogen recognition [2,4].

The correct assessment of the individual allelic diversity pattern and the underlying structural sequence variation is the basic requirement to understand the functional importance of MHC variability [5]. Multilocus MHC genes, however, pose great methodological challenges as inter-locus sequence similarity usually prevents the development of locus-specific primers. Thus, simultaneous amplification and genotyping of multilocus amplification products is often necessary e.g. [6-8].

Until recently, MHC genotyping was mainly done by cloning/Sanger sequencing or in species with low copy numbers by DNA-based methods using a gel matrix for allele separation, such as single strand conformation polymorphism (SSCP), denaturing gradient gel electrophoresis (DGGE) or reference strand-mediated conformational analysis (RSCA) combined with PCR reamplification of the separated bands (i.e. alleles) and Sanger sequencing. PCR amplification of multi-allelic templates and molecular cloning, however, have the disadvantage of a large error rate due to the formation of chimeras, i.e. amplicons that contain sequence motifs from two or more different alleles, and the formation of heteroduplexes, which become mosaic sequences through the DNA mismatch repair system during cloning [9,10]. As a consequence, gold standard rules to ascertain the assessment of correct levels of individual diversity have been progressively developed. These include simple modifications of PCR conditions, use of replicates, i.e. several independent PCR amplifications per individual, and sequencing of a large number of clones to reach allele saturation [10,11].

With the advent of next-generation sequencing (NGS) technologies, such as 454 GS FLX Titanium pyrosequencing (hereafter 454), it becomes now feasible to infer individual MHC allelic diversity with reasonable time as well as cost balance for larger sample sizes. The new approach has recently been applied to different non-model bird and mammal species with high copy number variation [8,12-18]. However, artefacts are still expected to be frequent. First of all, the error rate of 454 is not negligible, especially if the target sequence contains homopolymers [19], which can lead to repetitive errors. Moreover, since the sequencing of the targeted loci is still based on PCR amplification, some of the errors will be similar to those found in the traditional cloning/Sanger sequencing and can originate during the first target-specific PCR or the 454 sequencing procedures due to polymerase nucleotide misincorporation, chimera formation, or both. In particular, artefacts originating early in the process may be amplified across PCR cycles and therefore sequenced in multiple copies, which makes them difficult to identify. Chimeras may also be present in multiple copies, as they could be formed independently many times from the same sources during PCR cycles and resemble true alleles originating in vivo through recombination [10].

Unnoticed artefacts can lead to overestimation of individual allele numbers and overall diversity, crucial parameters for subsequent analyses on the functional importance of MHC diversity and the underlying selection processes. Even though including replicates in the 454 study design has been acknowledged as the most important source for detecting analytical errors, the percentage of implemented replicates in previous studies is null or rather low e.g. [12,14,15]. Gold standard rules are still in the developmental process for NGS data. Therefore, a rigorous 454 quality control is essential and strict approaches for data validation and allele calling are required to distinguish true alleles from artefacts [20,21].

Recently published studies e.g. [14-18] using 454 pyrosequencing data followed and modified the quality control and data validation protocols for MHC genotyping developed by Babik et al. [12], Galan et al. [13] and Zagalska-Neubauer et al. [8]. Babik et al. [12] validated variants on the basis of their frequency within individuals and considered variants with an observed frequency lower than a case-specific threshold as artefacts. They also validated variants based on their dissimilarity with the four most commonly found variants in a given sample and considered more distinct variants as more likely to be true alleles. Their approach paid no attention to the chimera problem, which are very dissimilar to both parent variants and might occur at a non-negligible frequency within individuals [13]. Galan et al. [13] developed a probabilistic model for determining the read coverage threshold T1 (minimum number of sequences per individual required for reliable genotyping) for validating individual genotypes at a given confidence level. Furthermore, Galan et al. [13] used a second threshold T2 (minimum frequency of a variant within an individual) for the minimum coverage required to define a variant as a true allele. Galan’s approach also considered the chimera problem and these artefacts were discarded after sequence alignment and BLAST procedures. Zagalska-Neubauer et al. [8] procedures included T1 as described in Galan et al. [13], but did not establish a second threshold for separating true alleles from artefacts. Instead, variants were accepted if they were present in at least two amplicons with a minimum of three reads (2-PCRs-3-copies-in-each rule) and were further checked for the possibility of representing artificial chimeras. Overall, the three analytical methods described above have several differences in their allele calling approaches but share at least two general assumptions: artificial sequences should be less frequent than true alleles; and artefacts should have their sources in the true alleles (e.g. chimeras and single base pair mismatches). They also mentioned that primers might have different specificity to different variants, but their analyses did not take differences in PCR amplification efficiency into account, i.e. differences in the probability that an allele is amplified due to primer mismatches. Ignoring differences in allele amplification efficiencies might have a significant effect on the read coverage required for reliable genotyping that is crucial for all subsequent downstream analyses and might cause an allelic dropout. Allelic dropout, i.e. alleles that are not detected in all individuals that biologically possess these alleles, might cause an artificial increase in the homozygosity values. Wrong genotypes and inflated homozygosities can bias the analysis of selection mechanisms in host-pathogen interactions and life history decisions such as mate choice, phenotype-genotype associations, recombination level, intra and inter-population differentiation, and associated conservation management decisions e.g. [10,22,23].

Here, we used 454 GS FLX Titanium pyrosequencing data from a wild rodent with high copy number variation in the MHC class II DRB locus to develop an allele calling workflow that can be transferred to any other non-model organism. Furthermore, we compared the results obtained by high-throughput pyrosequencing with the standard cloning/Sanger sequencing in all of our samples. Our workflow builds up on previous data processing and validation procedures [8,12,13], but has been considerably improved to identify mismatches occurring during the first PCR, the presence of chimeras, and the possibility of allelic dropout. It requires two amplicon replicates for all individuals, which allows the beginning of the classification procedure to be performed for each individual independently. Our approach presents two major differences to the three mentioned previous studies [8,12,13]. First, our method evaluates and classifies independently each single variant as either allele or artefact. Moreover, even though we also assume that artefacts are in general less frequent than true alleles, we do not rely on an arbitrary threshold to separate alleles from artefacts, in contrast to Babik et al. [12] and Galan et al. [13]. Unlike Zagalska-Neubauer et al. [8], we also do not make the strong assumption that identical variants should have one single classification. The second major difference to previous approaches is our a-posteriori analysis. We studied how differential allele amplification efficiencies influence the confidence level of genotypes and proposed, accordingly, a new way of estimating the minimum number of sequences per individual required for reliable genotyping. Moreover, we developed a method to assess the confidence level of genotyping a-posteriori per genotype, which helps to decide which alleles and individuals should be included in any further downstream analyses.

## Results

### Analysis of putative alleles and artefacts within the 454 dataset

**Figure 1:**
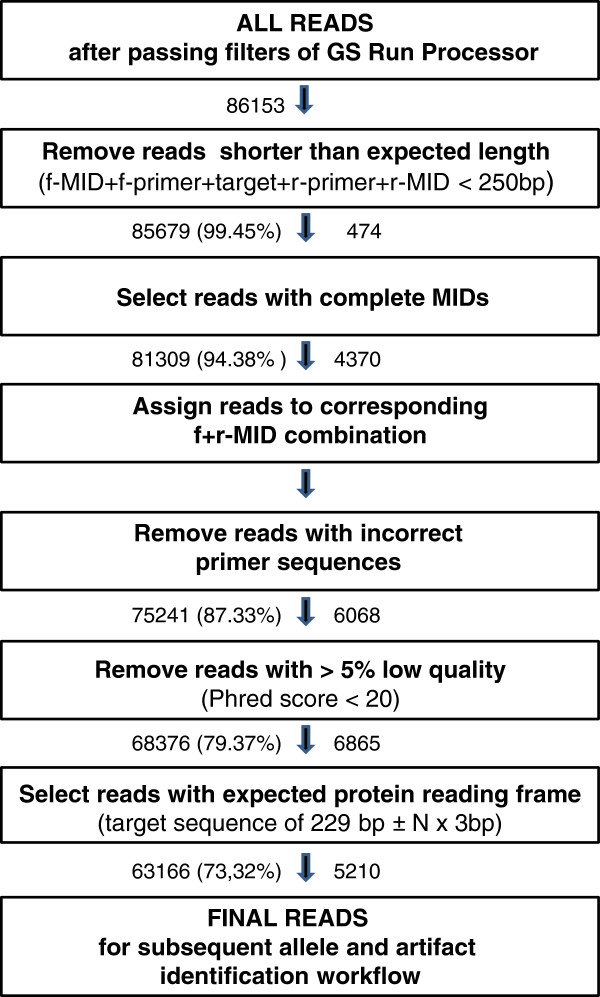
**454 reads filtering: pyrosequencing data quality assessment.** Pathway illustrating initial filtering steps to ensure data quality of reads obtained by 454 pyrosequencing and to facilitate the subsequent allele and artefact identification workflow. The number of reads included in a filtering step is indicated on the left of each arrow and the percentage of the initial number of reads in brackets. The number of discarded reads is shown on the right of each arrow.

We included all 40 individuals subjected to cloning/Sanger sequencing in duplicates (amplicon replicates) in one region (1/8th) of a 454 FLX Titanium picotiter plate. All 80 amplicons were barcoded (Additional file 1: Figure S1). All terms used to describe the subsequent results are outlined in Table 1. We obtained 86,153 sequence reads passing the filters of the GS Run Processor (Figure 1). 81,309 reads had the correct length with recognizable f-and r-MIDs, from which read numbers per amplicon ranged from 311 to 2276 (1028 ± 387) and from 1187 to 3193 per individual (summing both amplicon replicates). A total of 63,166 (73.3%) reads passed the initial filtering steps by showing the expected read length, complete primer sequences, high quality and no frameshifts (Figure 1).

**Table 1:** Definition of terms used

**Term used**	**Definition**
**Reads**	Sequences passing quality filtering criteria according to the standard amplicon pipeline from Genome Sequencer FLX System Software
**Cluster**	Set of identical reads within an amplicon
**Variant**	Specific sequence of a cluster
**Putative artefact**	Sequences or variants that are believed to result from polymerase or sequencing errors
**Chimera**	Reads containing sequence motifs from two different putative alleles
**Putative allele**	Variants that are believed to represent true alleles at the end of the allele and artefact identification workflow (Figure 2)
**Amplification efficiency**	Relative frequency at which an allele is amplified for a given primer pair
**Allelic dropout**	Alleles that are not detected in individuals that biologically possess those alleles
**1-2 bp diff**	1-2 base pair (bp) differences to the most similar variant with a higher frequency within an amplicon
**>2 bp diff**	>2 bp differences to the most similar variant with a higher frequency within an amplicon

We obtained reliable data for 36 out of the 40 individuals with 454 sequencing. Incongruencies between the two intra-individual amplicon replicates were detected for three individuals, probably due to either DNA cross-contamination or unnoticed exchange of barcodes before the first amplification. A fourth individual presented too few reads for one of the amplicons (20 reads after our filtering steps), even though we had targeted a very high number of reads per amplicon (>1000 in average). We removed these four individuals from subsequent analysis. For the remaining 36 individuals the number of reads after applying our initial data filtering (Figure 1) had an average per amplicon of 768 ± 314, ranging from 142 to 1600 reads per amplicon and from 291 to 2345 per individual.

**Figure 2:**
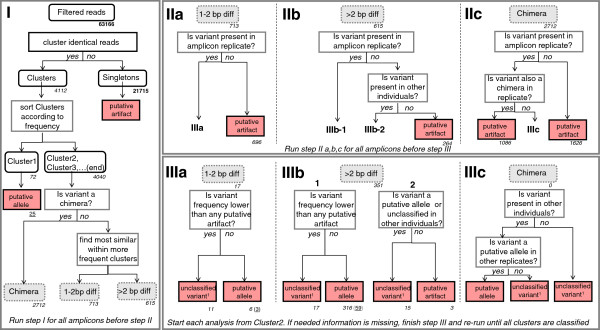
**Allele and artefact identification workflow: pathway for identification of artefacts and putative alleles.** Read numbers are given in bold, number of clusters are indicated in italics and the number of putative alleles is underlined. Note that putative alleles might be identified at different steps depending on the individual, explaining why the sum is larger than the total number of putative alleles observed in this study. Dashed gray rectangles indicate intra-amplicon cluster classifications (‘chimera’, ‘1-2 bp diff’, ‘> 2 bp diff’). Final cluster identification is highlighted in red. Unclassified variants^1^ include those neither classified as ‘putative artefacts’ nor as ‘putative alleles’ because they either appeared in a single amplicon or their frequencies were not above all artefacts. A detailed description of the workflow steps (I to III) is provided in the Method section.

**Figure 3:**
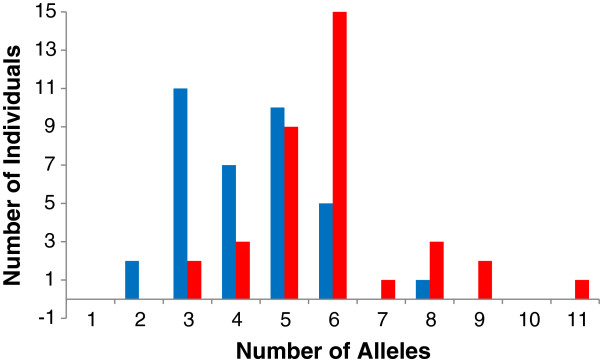
Differences in the individual number of alleles detected by cloning/Sanger (blue bars) and 454 pyrosequencing (red bars).

The subsequent workflow (Figure 2) allowed us to distinguish most of the filtered reads (63,091 out of 63,166 reads, 99.88%) into ‘putative artefacts’ (33,400 or ~53% of filtered reads) or ‘putative alleles’ (29,691 or ~47% of filtered reads). The remaining 75 reads were marked as ‘unclassified variants’, as they did not fulfil all assumptions to be called a ‘putative allele’ but could not be classified as ‘putative artefacts’. We checked the classification of each ‘unclassified variant’ among amplicons and detected three variants classified in at least one amplicon as a ‘putative allele’, but that were more often defined as ‘unclassified variant’ because of either lower frequency compared to ‘putative artefacts’ or absence in one individual’s amplicon replicate. The frequency inconsistencies of the latter variants suggest lower amplification efficiency when compared to other alleles. The presence of all three variants could be confirmed by Sanger sequencing after designing new allele-specific primers. We accepted those three variants as true alleles, but labelled them as ‘putative low efficiency alleles’. Those three alleles corresponded to two additional amino acid sequences (Desu-DRB*009, Desu-DRB*053). We found a total of 64 unique nucleotide true MHC variants, which translated into 57 putative MHC alleles on the amino acid level (Additional file 1: Figure S2). The ‘putative alleles’ were called MHC alleles for simplicity even though it was not possible to assign them to a particular locus. The alleles were denominated as Desu-DRB*X, where X corresponds to an allele number between 01 and 124 (GenBank under accession No’s KF134719-KF134782) according to the nomenclature (Klein et al. 1990). Without ‘putative low efficiency alleles’ between two and nine (5.4 ± 1.5) alleles were detected per individual, suggesting at least 5 copies of DRB in *Delomys sublineatus*. If the ‘putative low efficiency alleles’ are taken into account, the range of alleles detected by 454 pyrosequencing increases to 3-11 per individual (Figure 3).

**Figure 4:**
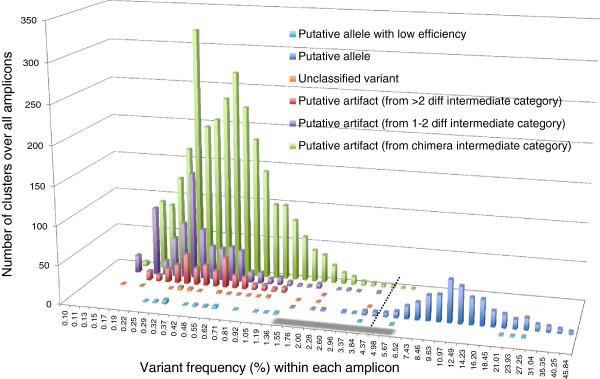
**Frequency distribution for all final cluster (= variant) classifications.** The cluster frequencies within their respective amplicons are indicated based on the final classification categories after the allele and artefact identification workflow. The frequencies on the x axis represent the proportion of reads within an amplicon (i.e. intra-amplicon frequency) that represent a given variant or cluster, while the y axis shows the total number of clusters over all amplicons for each intra-amplicon frequency range. The grey zone indicates the overlapping zone of artefacts and putative alleles, i.e. the zone between the most frequent variant classified as ‘putative artefact’ and the least frequent variant among the ‘putative alleles’ within an amplicon. The dotted line at 4.37% represents the threshold T2 according to Galan et al. [13] to separate putative alleles from putative artefacts. The most frequent artefact within an amplicon represented 5.4% of the total number of reads of this specific amplicon.

The variant frequencies within each amplicon (i.e. cluster frequencies) were analysed based on their classification categories of Figure 2. Figure 4 shows the prevalence of artefacts over alleles in the total number of clusters (artefactual clusters: 3674; putative allele clusters: 390). Although we confirmed only between 2-9 (3-11) alleles per individual (Figure 3), the number of variants varied between 6 and 126 (57 ± 26) per amplicon. The intra-amplicon classification for ‘putative artefacts’ was clearly dominated by 'chimeras' (37.7 ± 22.3) followed by ‘1-2 bp diff' (9.7 ± 10.5) and ' > 2 bp diff’ (3.7 ± 3) (see Table 1 for definitions). The most frequent variant classified as ‘putative artefact’ represented 5.4% of an amplicon and the least frequent variant among the ‘putative alleles’ represented only 1.5% of the total amount of reads within an amplicon (after excluding all intra-amplicon singletons).

### Comparison of individual MHC variability detected by 454 pyrosequencing to cloning/Sanger sequencing

In the 36 individuals investigated by both genotyping approaches, we detected 52 MHC alleles on the nucleotide level by conventional cloning/Sanger sequencing which could be considered as true MHC alleles according to the widely accepted standards in the literature. They corresponded to 49 unique amino acid sequences (Additional file 1: Figure S2). The average number of alleles per individual was 4.25 ± 1.34, and ranged from two to eight, corresponding to at least four DRB loci.

All alleles detected by cloning/Sanger sequencing were also detected by 454 pyrosequencing (Additional file 1: Figure S3) and showed a similar sequence variability pattern between the two methodologies, both at the nucleotide and amino acid levels (Additional file 2: Table S1). On the other hand, the NGS technology identified a further 12 alleles, and extended the genotypes of 31 out of the 36 individuals when compared to cloning results (Figure 3, Additional file 1: Figure S4). In addition, on the intra-individual level, 454 results indicate a significantly higher number of alleles than conventional cloning/Sanger sequencing (Figure 3, cloning: mean = 4.25 ± 1.34; 454-pyrosequencing: mean = 5.92 ± 1.65; Wilcoxon paired test: P < 0.001, N = 36), even though the number of alleles obtained by 454 pyrosequencing was significantly correlated to the ones obtained by cloning/Sanger sequencing (Additional file 1: Figure S4, rho = + 0.78, P < 0.001, N = 36).

### Allele amplification efficiencies

**Figure 5:**
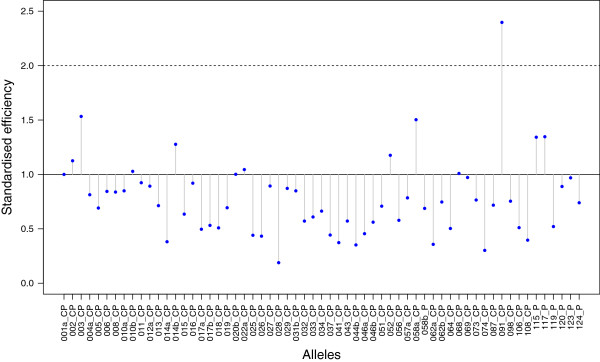
**Standardised allele amplification efficiency.** The amplification efficiency is estimated for each ‘putative allele’ by maximum likelihood and represented by a blue dot. Each dot is connected by a vertical line to the full horizontal line representing efficiency 1.0, which was defined using the first allele (Desu-DRB*001) as a reference (see text for details). The dashed horizontal line represents an efficiency of two, which could represent duplicated or homozygous alleles. ‘Low efficiency putative alleles’ are not represented (Desu-DRB*009a, Desu-DRB*053b, Desu-DRB*053c).

We used a maximum likelihood approach to estimate the amplification efficiency of each allele (see Methods) and found that there is a substantial variation in the amplification efficiency among alleles (Figure 5). The lowest amplification efficiency was reached for the allele Desu-DRB*028, which presented an amplification efficiency of 0.19, i.e. more than five times lower than the allele Desu-DRB*001 used as a reference (amplification efficiency = 1) (Additional file 2: Table S2). Maximum amplification efficiency was reached for the allele Desu-DRB*091, which with an efficiency of 2.40 is more than 12 times more efficient than Desu-DRB*028. No other allele presented an amplification efficiency close or equal to two, suggesting that Desu-DRB*091 was the only one corresponding to either a duplicated allele in different loci or a homozygous locus. This allele was present in four individuals always as the most frequent one (Cluster1) (Additional file 2: Table S2), and the ratio in frequency compared to the second most frequent cluster (Cluster2) ranged in average from 1.9 to 3.9 fold for these individuals, reinforcing the hypothesis that this allele is always present in duplicate. Even when omitting Desu-DRB*091, the span in efficiency between alleles represented an 8-fold increase between the least and the most efficient alleles.

### Effect of variation in amplification efficiency on threshold T1

The threshold T1 suggested by Galan et al. [13] (T1_Galan Negmult_, Table 2) aims to estimate the minimum number of reads necessary to obtain a reliable genotyping. Since Galan et al. [13] assumed that all alleles have the same amplification efficiency, we studied how T1 would be affected when taking variation in amplification efficiencies into account. To do so, we first developed a simulation procedure (T1_Galan Simul_) less computationally intensive but still highly comparable to T1_Galan Negmult_. T1_Galan Simul_ estimations and T1_Galan Negmult_ differed by less than one read on average and the biggest departure observed was three reads in the case where the presence of nine alleles was considered, which represents a relative error of only 3% (cf. last row in Table 2). The gain in computing time was substantial. For example, our simulation approach allowed us to obtain an accurate estimate of T1_Galan Simul_ for the case of nine alleles in less than three minutes using the easy but slow programming language R, while the efficient implementation programmed by Galan et al. [13] in the much faster language C++ requires several hours to provide a similar value.

**Table 2:** T1 thresholds

	**# Reads1**	**# Reads2**	**# Alleles**	**T1**_**Galan Negmult**_	**T1**_**Galan Simul**_	**T1**_**Simul VarAmplEff**_	**T1**_**Res1**_	**T1**_**Res2**_
**ID**	1186	306	5	50	50	74	62	66
102	418	326	6	62	61	92	176	64
153	329	205	6	62	61	93	111	121
161	416	382	4	38	38	77	113	80
165	252	524	5	50	50	128	240	137
190	295	418	6	62	61	138	102	139
211	285	443	4	38	38	63	155	69
215	506	829	6	62	61	64	64	59
223	410	335	5	50	50	100	88	87
225	458	383	4	38	38	58	53	52
248	239	463	6	62	61	68	67	71
252	536	549	5	50	50	73	61	65
256	409	297	5	50	50	74	117	93
266	341	342	5	50	50	86	105	57
272	317	317	5	50	50	53	53	69
281	430	324	7	75	74	120	111	177
347	171	261	6	62	61	83	96	73
430	300	288	4	38	38	62	57	43
449	545	283	4	38	38	82	91	75
492	133	1043	2	15	15	16	18	15
493	232	221	5	50	50	65	114	88
4695	361	701	6	62	61	113	142	120
4787	71	78	3	27	27	56	42	50
5092	273	535	6	62	61	98	75	111
5116	202	378	6	62	61	69	63	77
C3659	254	496	4	38	38	55	50	45
GO3120	220	456	5	50	50	203	93	314
GO3131	346	618	8	87	85	124	87	98
GO3132	511	794	4	38	38	51	54	53
GO3133	281	453	7	75	74	124	78	94
GO3134	363	678	8	87	85	124	102	342
GO3382	492	776	8	87	85	153	150	257
GO3394	482	609	3	27	27	28	25	25
GO3899	322	506	7	75	74	81	87	95
GO3922	235	359	6	62	61	75	68	136
GO3957	440	654	9	100	97	120	101	104

The threshold T1 defined as the minimum number of sequences per individual required for reliable genotyping was computed by Galan et al. [13] using a negative multinomial distribution (referred as T1_Galan Negmult_). We approximated the threshold by a much less computationally intensive simulation approach (T1_Galan Simul_). T1_Galan Negmult_ und T1_Galan Simul_ do not take differences in allele amplification efficiencies into account. We used a simulation approach to investigate the effect of variation in amplification efficiency between alleles on the minimum number of reads required to achieve a reliable genotyping (T1_Simul VarAmplEff_). Assuming that we oversampled each amplicon, we estimated what would have been the minimum number of reads that would have given us the same genotypes as obtained with our 454 data for the two amplicon replicates per individual by resampling (T1_Res1,_ T1_Res2_).

The new T1 values obtained assuming different amplification efficiencies (T1_Simul VarAmplEff_, Table 2) showed that taking variation in amplification efficiency into account leads to an increase of T1 by 1.03 to 4.06 fold (mean = 1.61) in order to maintain the same confidence level of genotyping. The highest sensitivity to the assumption of equal amplification efficiency concerned the genotype of individual GO3120, which consists of five alleles: the T1 value shifts from 50 to 203 when Galan’s assumption of equal amplification efficiency was relaxed. Interestingly, this genotype includes the allele Desu-DRB*028, which is the one with the lowest estimated amplification efficiency (Table 2, Additional file 2: Table S2).

**Figure 6:**
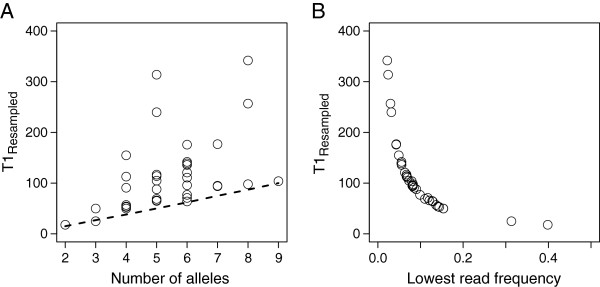
**Influence of the individual number of alleles (A), and the lowest read frequency (B) on the minimum number of reads required to determine a complete genotype with a 99.9% confidence level.** Each point represents a different individual (N = 36). The minimum number of reads required was a posteriori computed (T1_Resampled_) by resampling both amplicon replicates (T1_Resampled1_, T1_Resampled2_) and the maximal value was retained. The dashed line in **(A)** illustrates T1_Galan Simul_ computed according to Galan’s et al. [13] definition of an allele’s dropout (i.e. an allele associated with two reads or less; see text for details).

Subsequently, we further relaxed the assumption that amplification efficiency is fixed for each allele, by allowing each allele to have different amplification efficiencies in any given amplicon. Under these circumstances, T1 values (called T1_Resampled_), were between 0.93 and 6.28 fold (mean = 1.76) higher than T1_Galan Simul_ and between 0.76 and 2.76 fold (mean = 1.28) higher than T1_Simul VarAmplEff_ (Table 2). T1_Resampled_ increased with the number of alleles observed, as predicted by Galan et al. for T1_Galan Negmult_. Nonetheless, knowing only the number of alleles seems not to be sufficient to predict T1 accurately (Figure 6A; Spearman rank correlation between T1_Galan Simul_ and T1_Resampled_, rho = + 0.54, P < 0.001, N = 36). Figure 6B shows however that with the knowledge of the lowest observed read frequency present in a genotype, one could almost predict T1_Resampled_ to perfection (rho = − 0.996, P < 0.001, N = 36). Interestingly, the lowest read frequency can be predicted accurately (rho = + 0.79, P < 0.001, N = 36) from the ratio between the lowest allele amplification efficiency and the sum of amplification efficiency across all alleles constituting one genotype. Thus, we found that in our system T1_Resampled_ values can be predicted accurately from the lowest efficiency within a genotype (rho = − 0.80, P < 0.001, N = 36).

Therefore, we performed simulations to estimate T1 according to different values of the minimum amplification efficiency across all alleles (T1_Min Amp Eff_, Additional file 2: Tables S3 and S4). Additional file 1: Figures S5 and S6 show that T1_Min Amp Eff_ increases linearly with the number of alleles for a given value of minimum amplification efficiency considered. The results hold for T1_Galan Negmult_ (assuming an equal amplification efficiency of 1.0), as well as for any other value of minimum efficiency.

## Discussion

Although benefits of using NGS technologies in sequencing multilocus and highly variable regions such as the MHC are undeniable, these novel approaches are not exempt from biases and errors. While the recent literature puts emphasis on mistakes that can occur during the sequencing process, it is important to realize that long lasting limitations concerning genotyping can also occur because, similarly to the traditional cloning/Sanger sequencing procedure, NGS genotyping approaches are still based on amplification of the targeted locus via PCR. We showed that the diversity of artefacts emerging during the PCR step can largely outnumber variants corresponding to true alleles (in our study: artificial variants: 3674; putative allele variants: 390), and that the total number of sequence reads originating from artefacts can be higher than for true alleles. In addition, we demonstrated that PCR may be also responsible for allelic dropout caused by differential amplification efficiency between alleles. These two PCR-related side effects can lead to important mistakes at the level of allele calling and/or genotyping and therefore significantly alter downstream analyses relying on heterozygosity, allelic diversity, and genotype composition. The limitations caused both by PCR biases and errors and sequencing imply two main new challenges for NGS-based analyses: 1) the separation of true alleles from artefacts and 2) the correct evaluation of the reliability of genotypes obtained after allele calling, which should in addition to artefacts consider the possibility of allelic dropout. The methodology introduced in this study was specifically designed to address these problems.

### Separation of true alleles from artefacts

As shown in other MHC genotyping studies using 454 pyrosequencing e.g. [12,13], many of the variants obtained originate from artefacts. Artefacts may be produced by 454 pyrosequencing methodology during four different stages: the initial specific PCR, the emPCR, the sequencing reaction and signal processing. Although the use of an initial data quality check and reads filtering step (Figure 1) eliminates a great amount of them, a potentially large number of artefacts is still expected to remain, including many present in multiple copies within amplicons (in our study: total number of filtered reads: 63,166; artefacts: 33,400; artefacts occurring in multiple reads: 11,685,=18.2% of the filtered reads, Figure 2).

Our workflow was specifically designed to deal with the different kinds of artefacts potentially present. Homopolymer-associated errors along with other causes of insertion and deletion errors (i.e. indels) are the most common errors associated with 454 pyrosequencing base calling (i.e. sequencing signal processing). This type of error is identified and removed during our initial filtering steps, which eliminate reads with shifts in the reading frame. Chimeras caused by PCR artefact formation are the most frequent kind of artefact among the filtered reads and may be hard to detect because they usually resemble true recombining alleles [10]. However, we eliminated them by assuming that artefactual chimeras will be always present together with their sources (frequently true alleles) and in lower frequency. The basis for such assumption lies on the fact that chimera formation is thought to occur mainly on the last cycles of a PCR programme and would therefore be amplified less often than true alleles [10]. Artefacts can also originate from mismatches caused by polymerase errors during emPCR, which are eliminated to a large extent by deleting all singletons within amplicons. This kind of artefact usually corresponds to singletons because the probability that the same mismatch occurs independently in different reads should be extremely low [13], especially before position 400 bp of a 454-generated sequence [19]. Importantly, our workflow also allows the identification of mismatches caused by polymerase errors produced during library preparation (initial specific PCR) because individual amplicons were done in independent duplicates. While the probability of the same error occurring more than once during the initial specific PCR remains very low, implemented errors can be highly amplified when they occur in early PCR cycles [10] and the same amplified mistake can potentially be observed in multiple copies per amplicon. Therefore only the comparison of amplicon replicates allows the detection of polymerase errors.

The comparison of variants classification by our workflow to the ones obtained with alternative methods demonstrates that our method increases both the power (i.e. less false-negatives) and the robustness (i.e. less false-positives) of allelic assignments, as exemplified below. First, if we used the threshold T2 proposed by Galan et al. [13] to separate putative alleles from putative artefacts, all the different putative alleles defined by our allele and artefact identification workflow would still be recognized, but not for all individuals that carry them (i.e. false negatives). Precisely, we would use a T2 of 4.37%, which corresponds to the upper end of the artefact cluster frequency distribution within amplicons (Figure 4). The use of T2 would change the genotype composition for 12 individuals, with a total of 17 variants that would fail to be called ‘putative alleles’. In addition, ‘low efficiency putative alleles’ would be accepted for only two out of the 14 individuals we detected carrying this kind of allele.

Second, using the procedure suggested by Zagalska-Neubauer et al. [8], who accepted variants if they were present in at least two amplicons with a minimum of three reads (2-PCRs-3-copies-in-each rule), we would recognize all ‘putative alleles’ as well as our ‘low frequency putative alleles’ for all individuals, but their method would lead to erroneously consider as new ‘putative alleles’ two ‘putative artefacts’ and three ‘unclassified variants’ according to our classification (false positives). In addition, their method would fail to distinguish two ‘putative artefacts’ and two ‘unclassified variants’ that have identical sequences to true alleles in other individuals. We trust our classification because those specific variants were found either in low frequencies and/or were present in only one of an individual replicates. Finally, if we used a single amplicon for each individual like in their original approach, we would have immediately discarded 20 alleles that we detected in a single individual only.

Overall, distinguishing between true alleles and artefacts has been possible because our workflow combines several key features: First, using amplicon replicates for each individual facilitates variant classification and helps to recognize mistakes during laboratory procedures, which are more likely to occur with increasing numbers of multiplexed samples. Moreover, amplicon replicates help with the identification of alleles that are inconsistently amplified (allelic dropout) by a given primer pair. Second, our workflow does not rely on pre-defined thresholds based on intra-amplicon variant frequencies for identification of true alleles (such as T2), which could create a bias in allele calling by not detecting alleles with low or inconsistent intra-amplicon frequencies. Third, our workflow allows the identification of alleles with low amplification efficiency, indicating whether primers should be re-designed in order to cover these alleles/loci. Finally, our reads filtering pathway (Figure 1) and our allele and artefact identification workflow (Figure 2) can be adjusted to different needs in forthcoming MHC studies (see Additional file 3: Text S1).

### Comparison of individual MHC variability detected by 454 with cloning/Sanger sequencing

NGS technologies are likely to replace cloning and Sanger sequencing for MHC genotyping to a great extent in the near future. Nevertheless, traditional MHC genotyping approaches may remain an alternative to consider when a limited amount of samples is to be genotyped, as NGS is still expensive and many research groups do not have direct access to these technologies. Few studies compared the performance of one of the traditional methods with the outcome using a 454 approach using identical individuals [15,16]. However, this knowledge is essential to calibrate past and future findings. Our study has shown that results of 454 pyrosequencing and traditional cloning/Sanger sequencing are highly comparable at the qualitative level, but the NGS allowed us to detect a higher number of ‘putative alleles’. Additional comparative studies will shed light on whether this discrepancy between NGS and traditional methods is general or species-specific, and whether it is more pronounced in species with high copy numbers or not. We believe the higher allele detection probability of NGS builds on the higher sequencing depth that users are able to choose, rather than a real limitation of the traditional method. Consequently, cloning/Sanger sequencing could be used to supplement NGS studies, as long as the same T1 and allele calling workflow are used for both methods.

### Importance of allelic dropout: amplification efficiencies and confidence level of genotyping

Allelic dropout is an important source of bias and errors in allele calling or genotyping. The analysis of the relationship between allele amplification efficiency and the confidence level of genotyping (performed on variants classified as ‘putative alleles’) suggests that allelic dropout is a consequence of the low efficiency of certain alleles. Our workflow allowed us to identify three alleles (those classified as ‘low efficiency putative alleles’) very likely to undergo allelic dropout. Indeed, these alleles did not show a consistent frequency among the amplicons in all individuals that seem to carry these variants, were generally associated with low number of reads, and for a number of individuals these alleles were completely absent in one of the amplicon replicates.

We showed that low amplification efficiency does not only concern few alleles, but that many have amplification efficiencies lower than optimal. By assuming all alleles to have the same efficiency, the effective genotyping coverage obtained when following Galan et al.’s T1 recommendation becomes insufficient. Although we targeted an unusually high coverage and obtained numbers of reads much higher than Galan et al.’s recommendations, one ‘putative allele’ was still likely to be involved in allelic dropout. This allele was easily identified using our resampling-based method (the total number of reads did not meet our T1_Resampled_ for all amplicons presenting this allele). As we showed that the confidence level of genotyping was mainly constrained by the lowest read frequency among the alleles constituting the genotype of an individual, the problematic allele was logically the allele with the lowest amplification efficiency among all ‘putative alleles’ (Desu-DRB*028, standardised efficiency: 0.19). Consequently, this allele should not be included in the genotypes for downstream analysis because it is likely to suffer from allelic dropout in some individuals. Capturing the second least efficient allele (Desu-DRB*074) instead requires a number of reads that we obtained for all but two amplicon replicates. Nonetheless, these two problematic amplicons were associated with replicates that did reach the adequate T1 threshold value and did not include allele Desu-DRB*074, therefore eliminating chances of allelic dropout. Consequently, this second least efficient allele was reliably covered and could be included in further analysis using these individuals’ genotypes, as well as all alleles with a greater amplification efficiency value.

Overall, while our methodology successfully revealed instances of allelic dropout, differences in the expected number of loci found among individuals (one to five loci if we consider ‘putative alleles’ only, and two to six loci if we also consider the ‘low efficiency putative alleles’), suggests that other instances of allelic dropout have remained unnoticed. Therefore, it is likely that other alleles were completely missed during the first amplification unless this is explained by a variable number of MHC DRB loci within the same population [4].

A guideline to plan future experiments can be derived from the expected maximum number of alleles and the minimum amplification efficiency one could accept (e.g. 10 alleles and a minimum standardised amplification efficiency of 0.3). From these two numbers, information provided by Additional file 1: Figures S5 and S6 and Additional file 2: Tables S3 and S4 allow one to directly obtain the number of reads per amplicon required to reach 99.9% confidence level of genotyping. Note that because of the linearity of the relationship between T1 and the number of alleles (Additional file 1: Figures S5 and S6), one can derive graphically guidelines for higher number of alleles without any computational work. Importantly, the required number of reads per amplicon suggested by this method only provides guidance, but does not replace the coverage analysis based on resampling that has to be performed a-posteriori. This is because during the a-priori planning of a NGS project it is not possible to know the amplification efficiency across all alleles and we therefore assumed amplification efficiency to be optimal for all but the least efficient allele. Strong departure from this assumption may happen in certain system. Besides, the number of reads estimated to reach a certain confidence level of genotyping refers to reads that represent ‘putative alleles’ at the end of our workflow (i.e. after excluding all artefacts). In this study, we originally obtained 86,153 high quality reads from 1/8th of a 454 picotiter plate, and our final numbers after the initial filtering steps and having excluded artefacts included 29,691 reads which represented alleles, i.e. around one third of the initial reads. Estimating the required sequencing coverage per amplicon a-priori should probably consider similar high percentages of low quality reads and artefacts, or the possibility of increasing the coverage whenever necessary, and the coverage analysis based on resampling has to be performed a-posteriori after collecting the data in any case.

## Conclusions

Genotyping studies of multilocus MHC genes using NGS are prone to inaccurate allele-calling caused by both artefacts and unnoticed allelic dropout, especially due to the lack of matured approaches to deal with large amounts of data with an unknown level of complexity. At the same time, the correct assessment of an individual’s MHC constitution is the most fundamental pre-requirement to address the functional importance of MHC allelic diversity in evolutionary ecology, pathogen resistance and conservation. Our work, which builds on previous studies such as Babik et al. [12], Galan et al. [13] and Zagalska-Neubauer et al. [8], allows an efficient and robust evaluation of the allelic and genotyping coverage associated with a given set of primers. One of the crucial steps in our proposed workflow is the amplification of independent replicates for each individual, which overcomes some flaws from previous approaches, such as the misidentification of artificial sequences as true alleles, and the non-identification of allelic dropout and alleles with amplification deficiencies. Another crucial feature of our methodology is the consideration of allelic dropout via the measurement of the allele amplification efficiency. By ignoring variation in allele amplification efficiency, previous methodologies overestimated the confidence level of genotyping. In addition, we showed that amplification efficiencies can be used to estimate the minimum number of reads required for genotyping. Allelic dropout cannot be avoided easily but it does not represent a major problem as long as alleles/loci that might be affected by this phenomenon are identified and removed from the downstream analysis whenever appropriate. Combining our workflow with the study of the impact of differences in amplification efficiency offers the chance for researchers to evaluate and understand data generated by NGS in great detail, improving confidence over the approach as well as the follow-up analyses and subsequent applications.

## Methods

### Ethics statement for field work and collecting DNA samples

For the present study, we used 40 unrelated samples of the Pallid Atlantic Forest Rat *Delomys sublineatus* (Thomas, 1903), an endemic species in the Brazilian coastal rainforest Mata Atlantica. This rodent is used as an indicator species in conservation studies [24-32]. The species was selected due to its high copy number variation in MHC class II DRB loci and relevance for further immuno-ecological studies [32-34]. Trapping, animal handling and collection of tissue samples for this project complied with international guidelines and were approved and permitted by the national authority, the Instituto Brasileiro do Meio Ambiente e dos Recursos Naturais Renováveis-IBAMA (permission 11573-2) and conformed to guidelines sanctioned by the American Society of Mammalogists Animal Care and Use Committee [35]. Because our study did not involve any experimentation (e.g. maintenance in captivity, injection of drugs, or surgery) an approval from the Ethics Committee of the Institute of Biosciences, University of São Paulo (Comissão de Ética em Uso de Animais Vertebrados em Experimentação, CEA, http://ib.usp.br/etica_animais.htm) was not required.

### Traditional approach: molecular cloning followed by Sanger sequencing

Genomic DNA was extracted from ear tissue using the GEN-ial all tissue kit (GEN-IAL GmbH, Troisdorf, Germany) following the manufacturer’s instructions. We examined a 228 bp fragment of MHC class II DRB exon2 coding for the major part of the functional important antigen-binding sites of the β1 domain. The targeted fragment was amplified in all individuals (N = 40) using the primer pair JF8-iV (f-primer: 5'-TGGACGAGCAAGACGTTCCTGT-3') and Tub2JF (r-primer: 5'- CGAYCCCGWAGTTGTGTCTGCA-3'). The primers fit to usually highly conserved parts of MHC class II exon2 across mammals. The forward primer has been designed to optimize specificity to *Delomys sublineatus*. Extensive testing of different primer combinations have been carried out at the beginning of the project to design the best primers possible. To minimize misincorporation errors, PCR products were generated with a proofreading polymerase (HotStar HiFidelity polymerase, Qiagen, Hilden, Germany). Two independent PCRs for cloning were performed per individual and the outcome was considered as congruent if the identical number and sequence pattern of variants were detected by subsequent Sanger sequencing.

PCR was conducted in a total reaction volume of 20 μl including 200 ng DNA, 0.75 μM of each primer (Sigma-Aldrich, Steinheim, Germany), 5× HotStar HiFidelity PCR buffer (including dNTPs and MgSO4) and 0.5 unit of *Taq* polymerase. Thermocycling comprised an initial denaturation step at 96°C for 10 min, followed by 33 cycles of 1 min denaturation at 96°C; 1 min annealing at 58°C and 3 min extension at 72°C. A final extension step was performed at 72°C for 15 min. PCR products were purified (QIAquick PCR Purification Kit, Qiagen, Hilden, Germany) and cloned into a pCR®4-TOPO vector using the TOPO TA cloning kit for sequencing (Invitrogen, Karlsruhe, Germany). Initially, up to 90 clones were sequenced per individual to detect the saturation threshold. The relationship between the number of different MHC alleles in relation to the number of sequenced clones per individual indicated that the saturation plateau was reached after 20-25 clones in most of the individuals (Figures not shown). As a conservative approach, 40 recombinant clones per individual were selected and amplified using the vector primers T7 and M13 rev. Cloned PCR products were purified and sequenced directly on an A3130*xl* automated sequencer using the BigDye Terminator v3.1 Cycle Sequencing Kit (both Applied Biosystems Deutschland GmbH, Darmstadt, Germany).

The criteria used to define a sequence from cloning/Sanger sequencing as a true MHC allele were based on its occurrence in at least two independent PCR reactions derived from the same or different individuals. Allele sequences were named according to the nomenclature rules set by Klein et al. [36].

### NGS approach: laboratory procedures for 454 pyrosequencing

In order to facilitate PCR error and bias recognition, all individuals (N = 40) were amplified twice in independent PCRs (referred to as amplicon replicates), i.e. each individual was included twice on the 454 plate using different barcoding tag combinations. For library preparation we used fusion primers consisting of four parts (Additional file 1: Figure S1). At the 5’ end the primers contain an adaptor sequence (forward adaptor A: CGTATCGCCTCCCTCGCGCCA, reverse adaptor B: CTATGCGCCTTGCCAGCCCGC), followed by an internal library key (TCAG). A combination of barcodes sequences recommended by Roche, the so-called multiplex identifiers (MIDs, 10 bp long), were used to identify each amplicon replicate. For each direction a set of nine different MIDs differing in at least 3 positions from each other was chosen (f-MID, r-MID). The 9 forward and 9 reverse MIDs produce 81 possible combinations, and allowed us to pool and distinguish 80 different samples (two PCR replicates for each of the 40 individuals) in one single 454 region by using only 18 different fusion primers. The DRB-specific amplification primers were the same as the ones used for cloning (JF8-iV, Tub2JF) and formed the last part of the fusion primers (Additional file 1: Figure S1).

PCR was carried out in 25 μL reaction volumes containing 0.4 μM of each fusion primer, 0.2 mM dNTPs, 2.5 μL FastStart buffer and 1.25 U FastStart HiFi Polymerase (Roche Diagnostics GmbH, Grenzach-Wyhlen, Germany). Reactions comprised an initial denaturation step at 94°C for 2 min, followed by 35 cycles of 30 sec denaturation at 94°C; 30 sec annealing at 58°C, 1 min extension at 72°C and a final extension at 72°C for 7 min. After amplification the PCR products were purified using the Agencourt AMPure system (Agencourt Bioscience Corporation, Beverly, MA) and then quantified by the Quant-iT PicoGreen dsDNA Assay Kit (Invitrogen Corporation). Subsequently, all amplicons were diluted to 200,000 molecules/μl and pooled.

During emulsion PCR (emPCR), the library was clonally amplified using the GS FLX Titanium SV emPCR Lib-A kit (Roche Diagnostics GmbH). Following immobilization onto DNA capture beads, the bead-bound amplicons were added to the emulsion oil and the amplification reagents. Through shaking on a Tissue Lyser (Qiagen) each bead is captured within its own microreactor where PCR amplification occurs. EmPCR was dispensed into a 96-well plate and the PCR program was run according to manufacturer's instructions. After amplification, the beads were recovered by emulsion breaking and washed. Using a biotinylated primer A (complementary to adaptor A), which is bound to streptavidin-coated magnetic beads, DNA library beads were enriched. The DNA library beads were then separated from the magnetic beads by melting the double-stranded amplification products, leaving a population of bead-bound single-stranded template DNA fragments, to which the sequencing primer was annealed. Then the library pool was sequenced in one GS FLX Titanium run on 1/8th of a 70 × 75 PicoTiter plate.

### Initial 454 data quality check and reads filtering

454 sequencing images and signals were processed with the Genome Sequencer FLX System Software using the standard amplicon pipeline option. A definition of all terms used is provided in Table 1. In order to ensure data quality and to select reads for subsequent data validation procedures we used several filtering steps (Figure 1). As the initial step, all reads substantially shorter (<250 bp) than the expected length (~290 bp including MIDs, specific MHC primers and target) were removed. It has to be noted that at this step potential pseudogenes with structural abnormalities were lost which might be of interest to population genetic studies or to comparative genetics. Subsequently, all remaining reads were sorted based on their forward and reverse MID, discarding those reads with incomplete/incorrect MID sequences which could not be assigned to any individual. From this point onwards all steps were performed for each independent amplicon (i.e. each MID combination) separately. Reads were further filtered out when showing an incomplete/incorrect primer region and/or lower quality (less than 95% bases with Phred quality score > 20). In the last steps we looked for indels, allowing only multiple of 3 bp indels (corresponding to one amino acid), and selected all reads with an expected target sequence length. The reads were finally aligned with Muscle v3.8 [37] using a Python script to automate the process. The alignments were entered in the software Geneious Pro v5.5 [38] and a visual inspection was quickly performed in order to identify possible reads with changes in the reading frame. A shift in the reading frame was not accepted because the region analyzed comprises a coding exon. All remaining reads were ready for subsequent allele and artefact identification workflow to detect potential sequencing errors and PCR artefacts before the final putative allele calling (Figure 2).

### Putative MHC alleles and artefacts identification

A workflow consisting of a series of steps (I to III) was developed for assigning the final reads to putative MHC alleles (Figure 2). Each step was performed for all amplicons across individuals before moving to the next step, and step III was repeated until all clusters were classified either as ‘putative artefact’, ‘unclassified variant’, or ‘putative allele’.

The first step of this workflow treats each independent amplicon separately and begins with the assembling of all identical reads into clusters (step I). All reads not assigned to clusters (i.e. singletons) were considered as ‘putative artefacts’ and not included in further analyses as they are likely a result of PCR or sequencing errors. For the subsequent steps all clusters (from now on also called variants as they represent a specific sequence) were numbered and organized in hierarchical order based on their frequency (i.e. Cluster1 as the most frequent). The classification of variants at the end of step I was done based on an intra-amplicon evaluation, and assigned the clusters to three different categories: ‘chimera’, ‘1-2 bp diff’, ‘> 2 bp diff’. This classification was done to facilitate subsequent artefacts recognition, based on two important assumptions: 1. Artefactual sequences generated in vitro are less frequent than their source(s) (usually true alleles) within an amplicon and 2. Artefacts should be less frequent within an amplicon than any true allele. With these assumptions, we could work with variant frequencies (i.e. percentages within the amplicon) as the main tool for defining ‘putative alleles’. According to these assumptions, true MHC alleles were expected to be amongst the most frequent clusters in the dataset, although some attention must be paid for possible amplicons for which primers presented a sub-optimal efficiency and real MHC alleles might therefore appear in lower frequencies.

Chimeras were identified with a dedicated Python script, which simply tested if the query cluster could be a combination of two different, but more frequent clusters. Although it could be difficult to differentiate in vitro chimeras from true recombinants, we have considered that chimeras should always be less frequent than the putative alleles from which they originated, as stated in our assumption number 1 (see above).

After sorting out probable chimeras, all remaining clusters (starting with Cluster2) were compared with more frequent ones, in order to identify the most similar variant. The clusters were assigned to two intra-amplicon categories when compared to their most similar cluster: either one or two base pair differences (‘1-2 bp diff’) or more than two base pair differences (‘>2 bp diff’). ‘1-2 bp diff’ are likely to be polymerase errors that got amplified during PCR, and ‘>2 bp diff’ probably represent more complex kinds of artefacts that are hard to be defined, such as mosaics of more than two fragments or chimeras plus polymerase errors (we have found examples of both kinds). After finalizing step I for all individuals, all data was organized in a local PostgreSQL database (http://www.postgresql.org/) in order to facilitate the subsequent comparisons and to keep all information organized and promptly available.

The second step (step II) of the workflow (Figure 2) aims to recognize most of the putative artefacts, by comparing both MID combinations (i.e. amplicon replicates) of each individual, as well as amplicons from different individuals. It begins with checking for each intra-amplicon cluster classification the occurrence of a given variant in the second amplicon. The absence of variants labelled as ‘1-2 bp diff’ (IIa) or ‘chimera’ (IIc) in the second amplicon classifies those as ‘putative artefacts’. The same is not true for ‘>2 bp diff’, which is classified as ‘putative artefact’ at this step only if it is not present in any other individual. Finally, whenever a variant is classified in both amplicons as a chimera, it is also classified as a ‘putative artefact’ (IIc).

All the ‘putative artefacts’ classified until now will be used in step III to help defining ‘putative alleles’, based on their intra-amplicon frequencies. Variants grouped into the categories ‘1-2 bp diff’ (IIIa) and ‘>2 bp diff’ (IIIb) which were present in both amplicon replicates but less frequent than any annotated artefact were labelled as ‘unclassified variants’, since they are not amongst the most frequent clusters but are unlikely to be artefacts if they appear in both replicates in at least two copies each (i.e. in clusters). Those present in both amplicons and more frequent than all annotated artefacts are considered as ‘putative MHC alleles’ (IIIa, IIIb-1). Variants labelled as ‘>2 bp diff’ not detected in the second amplicon were further checked for presence in other individuals (IIIb-2). If the variant is considered as a ‘putative allele’ or ‘unclassified’ in another individual it was labelled as ‘unclassified variant’, otherwise it was considered as a ‘putative artefact’ (IIIb-2). In our analysis, none of the chimeras were grouped to a different category in the amplicon replicate, and therefore all were considered as ‘putative artefacts’. We have, however, designed further classification steps for those cases where variants do not appear as ‘chimeras’ in both replicates. In this case, a variant will be assumed to be a ‘putative allele’ if it is present in other individuals, and will be considered as a natural recombinant.

At the end of step III, all variants are classified either as ‘putative alleles’, ‘putative artefacts’ or ‘unclassified variants’ (Figure 2) for each amplicon in which they occur. Variants classified as ‘putative alleles’ and/or ‘unclassified’ were further evaluated in order to identify alleles with inconsistent frequencies among amplicons. Variants recognized as ‘putative alleles’ in some individuals but more frequently classified as ‘unclassified variants’, because of low frequencies (compared to artefacts) or absence in one of the individual amplicons, were identified as ‘low frequency putative alleles’.

### Alternative ready-to-use tools

Most of our analysis was done without the use of specifically designed software packages. All data were organized in a PostgreSQL database and analyses were mainly done with either self-coded Python scripts or SQL queries. However, there are a number of software packages available that facilitate following our workflow (Figure 2) without the need of coding. The jMHC software [39] and SESAME (SEquence Sorter & AMplicon Explorer) [40], for example, allow 454 filtered data to be separated by barcode (including 2-sided barcodes) and summarizes all information about variables present in one or more amplicons. Alignments for single amplicons ordered based on variant frequency can be saved in single files. Visualization of alignments for chimera identification may be done with MEGA5 [41] for instance, as well as the construction of pair-wise sequence comparison matrices, useful to distinguishing closely related sequences (1-2 bp) from more unrelated ones (>2 bp). All data originating from jMHC can be imported in a table format and organized using commonly available spreadsheet softwares. Any extra information (e.g. intra-amplicon and inter-amplicon analysis) may be entered in the same table, which continues to be incremented as one moves forward throughout our workflow (Figure 2).

### Estimation of allele’s amplification efficiency

The reliability of the coverage of genotypes depends on allele amplification efficiencies. We derived a maximum likelihood optimization approach allowing the estimation of the relative amplification efficiency of each allele. Importantly, this method assumes that each allele can be characterized by a single efficiency value i.e. that the amplification efficiency of a given allele is 1) independent from the genotype and 2) similar among PCR samples with identical conditions. The method uses the density function of the multinomial distribution that provides the probability of an event that would have led to the number of reads observed for a given amplicon and for a given set of amplification efficiency values. Multiplying the probabilities associated with each amplicon over the entire dataset (or summing them on a log-scale), one can therefore obtain the (log)likelihood of the data given the amplification efficiency considered. Since the (log)likelihood is a function of the amplification efficiency of each allele, one can estimate the amplification efficiencies maximizing this function using an optimization algorithm. It provides amplification efficiency values that are the most likely to have resulted to the observed dataset. The method has been implemented in the language R [42], a free open source statistical program, and the script is provided and detailed in additional material (Additional file 4: R-Codes).

The estimated amplification efficiencies obtained by this process are relative values. Consequently, we then estimated the standardised amplification efficiency for each MHC allele using the amplification efficiency of MHC allele Desu-DRB*001 (Additional file 1: Figure S2, Additional file 2: Table S2) as reference, i.e. considering its standardised amplification efficiency to be one. This allele was chosen as the reference because the MHC forward primer was developed based on the DNA sequence of a longer fragment obtained originally with a pair of degenerated primers and that corresponds to Desu-DRB*001. The reverse primer used in this study remained degenerated. To obtain standardised amplification efficiency values, we therefore recomputed the amplification efficiency of other alleles by dividing their relative amplification efficiency by the efficiency of the reference allele. Importantly, this standardization is necessary for identifying potentially duplicated alleles (i.e. those with a standardised efficiency ≥ 2) but it plays no role in the study of the variation in amplification efficiency between alleles, nor for coverage analyses described below.

### Estimation of the minimum number of reads needed-estimation of Galan’s T1 considering equal and different allele amplification efficiencies

To compare the minimum number of reads needed to reach a certain coverage under the assumption of equal amplification efficiencies between alleles (T1_Galan Simul_) and taking variation in amplification efficiency into account (T1_Simul VarAmplEff_), we used a simulation approach based on random drawings in a multinomial distribution. In both conditions, we estimated for each genotype the minimum number of reads so that all alleles were represented by at least two reads in 99.9% of 10,000 simulations. We replicated the estimation of T1 100 times for each genotype and took the median values to generate T1_Galan Simul_ and T1_Simul VarAmplEff_ (Additional file 4: R-Codes).

To evaluate our previous assumption that the amplification efficiency of a given allele is independent from the individual and genotype, we allowed each allele to have a different probability in different amplicons. To do so, we used the allele frequencies observed in each amplicon as the expected amplification efficiencies (i.e. number of reads representing one allele divided by the sum of all reads representing alleles in one single amplicon). We simulated the distribution of the number of reads among alleles for each genotype by performing a random sampling with replacement of the observed reads and estimating T1_Resampled_ as the number of reads sampled required for reaching the threshold for genotype coverage. For each genotype we performed 1000 simulations per number of reads and T1_Resampled_ was estimated as the lower number of simulated reads which identified all putative alleles in at least 99.9% out of the 1,000 simulations. Again, this computation was performed 100 times for each genotype and the median of the T1_Resampled_ values was retained. The entire procedure was performed on both amplicon replicates of each individual. The dedicated R script is provided in the Additional file 4: R-Codes.

Finally, we estimated the required number of reads for different values of minimal amplification efficiency and number of alleles. To do so, we simulated number of reads for a given number of alleles by assuming that the amplification efficiency of all but one allele is equal to one. For the remaining alleles, we set the amplification efficiency to the minimum value investigated and considered for this latter all values between 0.01 and 1. T1 was computed 100 times for each number of alleles and minimum amplification efficiency and 10,000 sets of reads were simulated for each run. The final T1 value considered was again the median value among the 100 T1 values computed for each combination of number of alleles and minimum amplification efficiency.

## Competing interests

The authors declare that they have no competing interests.

## Authors’ contributions

SS initiated the overall study, supervised the sample collection in Brazil and the cloning and Sanger sequencing performed by student assistants and technicians, she did the cloning and Sanger sequencing data processing and performed the comparison between NGS and Sanger sequencing. AC designed, programmed, and carried out the allele amplification efficiency analysis. CJM supervised the NGS molecular biology work performed by technicians, carried out NGS data processing, and designed and programmed the allele calling workflow. All authors drafted and approved the final manuscript.

## Supplementary Material

Additional file 1: Figure S1 Describes the fusion primers and barcode combinations for 454 library preparation. **Figure S2** contains an alignment of amino acid sequences of *D. sublineatus* MHC class II DRB alleles detected by cloning/Sanger sequencing and/or 454 pyrosequencing and outlines antigen-binding sites [43]. **Figure S3** shows the allele frequencies in individuals genotyped by cloning/Sanger sequencing and 454 pyrosequencing. **Figure S4** indicates the comparison of levels of individual MHC class II DRB diversity obtained by conventional cloning/Sanger sequencing and next-generation 454 pyrosequencing. **Figure S5** outlines the predicted minimum number of reads (T1_Min Amp Eff_) required to determine a complete genotype for at least two reads per allele (99.9% confidence level). **Figure S6** shows the same as Figure S5 but for at least three reads per allele. Click here for file

Additional file 2: Table S1 Shows the MHC-DRB diversity in *D. sublineatus* detected by cloning/Sanger sequencing and 454 pyrosequencing using the identical individuals. **Table S2** shows a list of the standardised allele amplification efficiencies. **Table S3** shows the predicted minimum number of reads necessary to obtain at least two reads per allele based on minimum amplification efficiency. **Table S4** shows the predicted minimum number of reads necessary to obtain at least three reads per allele based on minimum amplification efficiency. Click here for file

Additional file 3: Text S1 Describes possible adjustments of the reads filtering pathway (Figure 1) and our allele and artefact identification workflow (Figure 2) to different needs in forthcoming MHC studies. Click here for file

Additional file 4 **The R-Codes used to estimate the allele’s amplification efficiency and the different T1 thresholds.** Click here for file
