# Supplementary material for: MHC genotyping of non-model organisms using next-generation sequencing: a new methodology to deal with artefacts and allelic dropout
Source: BMC Genomics. 2013 Aug 9;14:542. doi: 10.1186/1471-2164-14-542 (PMC3750822; doi:10.1186/1471-2164-14-542)
Supplement: Additional file 1: Figure S1 — Describes the fusion primers and barcode combinations for 454 library preparation. Figure S2 contains an alignment of amino acid sequences of D. sublineatus MHC class II DRB alleles detected by cloning/Sanger sequencing and/or 454 pyrosequencing and outlines antigen-binding sites [43]. Figure S3 shows the allele frequencies in individuals genotyped by cloning/Sanger sequencing and 454 pyrosequencing. Figure S4 indicates the comparison of levels of individual MHC class II DRB diversity obtained by conventional cloning/Sanger sequencing and next-generation 454 pyrosequencing. Figure S5 outlines the predicted minimum number of reads (T1Min Amp Eff) required to determine a complete genotype for at least two reads per allele (99.9% confidence level). Figure S6 shows the same as Figure S5 but for at least three reads per allele. [file 1471-2164-14-542-S1.docx]

**Additional data file 1 contains supplementary Figures S1 to S3.**

**Figure S1**

**A**


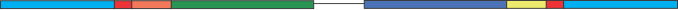


Adaptor A

Key

f-MID

f-primer

r-primer

r-MID

Key

Adaptor B

target

**B C**

| **f-MID** | **Sequence** | **r-MID** | **Sequence** |  |  | **A001** | **A002** | **A003** | **A004** | **A005** | **A006** | **A007** | **A008** | **A009** |
| --- | --- | --- | --- | --- | --- | --- | --- | --- | --- | --- | --- | --- | --- | --- |
| A001 | ACGAGTGCGT | B001 | TCTCTATGCG |  | **B001** | 153 | 5116 | GO3131 | GO3957 | 347 | C3659 | 4695 | GO3133 | GO3899 |
| A002 | AGACGCACTC | B002 | TGATACGTCT |  | **B002** | 165 | 87 | GO3132 | 211 | 430 | Go3922 | 102 | GO3134 | 256 |
| A003 | CGTGTCTCTA | B003 | TAGAGACGAG |  | **B003** | 190 | 179 | 492 | 248 | 161 | 225 | GO3120 | 5056 | 266 |
| A004 | CTCGCGTGTC | B004 | TCGTCGCTCG |  | **B004** | 195 | 214 | 493 | GO3394 | 223 | 272 | 5092 | GO3382 | 281 |
| A005 | TCTCTATGCG | B005 | ACATACGCGT |  | **B005** | 449 | 215 | 4787 | 252 | 153 | 5116 | GO3131 | GO3957 | 347 |
| A006 | TGATACGTCT | B006 | ACGCGAGTAT |  | **B006** | C3659 | 4695 | GO3133 | GO3899 | 165 | 87 | GO3132 | 211 | 430 |
| A007 | CATAGTAGTG | B007 | TGTACTACTC |  | **B007** | Go3922 | 102 | GO3134 | 256 | 190 | 179 | 492 | 248 | 161 |
| A008 | CGAGAGATAC | B008 | ACGACTACAG |  | **B008** | 225 | GO3120 | 5056 | 266 | 195 | 214 | 493 | GO3394 | 223 |
| A009 | ATACGACGTA | B009 | CGTAGACTAG |  | **B009** | 272 | 5092 | GO3382 | 281 | 449 | 215 | 4787 | 252 |  |

**Figure S1:** Fusion primers and barcode combinations for 454 library preparation. **A** shows the fusion primers consisting of four parts. Forward fusion primers consisted of the adaptor sequence A, an internal library key (TCAG), a combination of barcodes sequences (multiplex identifiers, f-MIDs), and the f-primer. Reverse fusion primers consisted of the adaptor sequence B, an internal library key (TCAG), a combination of barcodes sequences (multiplex identifiers, r-MIDs), and the r-primer. **B** indicates the barcode sequences. **C** shows the barcode combinations per individual.

**Figure S2**

Position 3 9 11 13 19 23 28 31 33 37 43 48 53 57 60 63 66 70 73 77

ABS ------*-*- *--------- -----*-*-*- ---**----- ---*-*---* *---*--*-* *--*---*-- *---*

ConsSites -*-**----- -******-** -**-*-*---* -**---**** -**-***-** **------** -*---*--** -**--

Desu-DRB*001CP SAARFTWQGK AECHFENGTE RVRLLARLFYN REEYVRFDSD LGEFRAVTEL GRPDEEYFNS QKDYMEQMRA AVDR-

Desu-DRB*002CP .....LEYL. F.....N... ...Y.V.DI.N Q..V...... V......S.. ..STA...N. R..FL.DL.. R..TI

Desu-DRB*003CP .....LV.D. S.....N... ...Y.L.Y..N ...F...... V..Y...... ........N. ...LL..R.. K..TY

Desu-DRB*004CP .....MEYV. H.....N... ...Y.Y.DI.N ....L..... V......... ........N. R.EIL.EK.. K..TY

Desu-DRB*005CP .....LEHV. F.....N... ...F.V.DI.N ...V...... V......... ........N. R..FL.E... G..K-

Desu-DRB*006CP .....LEYV. Y.....N... ...Y.E.VV.N ...F...... V......... ...IAKDWN. R..LL.DR.. Q..TV

Desu-DRB*008CP .....MESL. S.....N... .....Q.YI.N .......... V......... ..LS...YN. R.E.I.DL.. ...TF

Desu-DRB*009CP .....LEYV. H.....N... .....Q.H..N ...FLL.... V..Y...... ...E...YN. R.EIL.R... E..K-

Desu-DRB*010CP .......... G.....N... S.........N .......... V......... .....K.WN. ..E.L..... ....-

Desu-DRB*011CP .....MEYST S.....N... ...Y.D.Y..N ...F...... V..Y...... ...E...WN. R..LL.EK.. K..TY

Desu-DRB*012CP .....MEYV. H.....N... ...F.Y.DI.N ....L..... V......... ........N. R..LL.EK.. K..TY

Desu-DRB*013CP .....LKHV. H.....N... ...Y.Y.DI.N ...FA..... V......... .......WN. ...LL.R... E..K-

Desu-DRB*014CP .....MESL. S.....N... ...F.E.Y..N Q......... V..Y...... ..K..KNWN. R..LL.RK.. E..TY

Desu-DRB*015CP .......... G.....N... S.........N .......... V......... .......WN. ..E....... ....-

Desu-DRB*016CP .....LV.T. H.....N... ...H.E.Y..N ...F...... V..Y...... ..K..KNWN. ..E.L.DA.. ...TY

Desu-DRB*017CP .......... ......N... ..........N .......... .......... .......WN. ..E....... ....-

Desu-DRB*018CP .....MEYV. Y.....N... ...Y.E.VV.N ...F...... V......... ...IAKDWN. R..LL.DR.. Q..TV

Desu-DRB*019CP .......... G.....N... S.........N ...F...... V......... .....K.WN. ..EIL..... ....-

Desu-DRB*020CP .......... ......N... ..........N .......... .......... ..R....WN. .......... ....-

Desu-DRB*022CP .....MEYV. H.....N... ...F.Y.DI.N ....L..... V......... ........N. R.EIL.EK.. K..TY

Desu-DRB*025CP .....LNYI. F.....N... ...F.H.DI.N ....L..... V..Y...... ..Q..KDYN. ..E.L.GA.. ...TY

Desu-DRB*026CP ..D..ML... ......N... ..........N ...F...... .......... .......WN. ..E....... ...TY

Desu-DRB*027CP F......... ......N... ..........N .......... V......... .......WN. ..E....... ....-

Desu-DRB*028_P .....LEHV. F.....N... ...Y.E.VV.N .......... V..Y...... .....KNWN. ...LL.DR.. S..TY

Desu-DRB*029CP .....LKYV. S.....N... ...Y.Y.DI.N ...F...... V......... .......WN. R.E.L.DA.. ...K-

Desu-DRB*031CP .....LECV. H.....N... ...Y.E....N ...FA..... V......S.. .......WN. ...LL.RK.. E..TY

Desu-DRB*032CP .....L.... ......NR.. ...F.E.YI.N .......... V..Y...... .......YN. R.EIL.EK.. E..TY

Desu-DRB*033CP .....MESL. S.....N... ...F.E.Y..N ...V...... V......... ..K..KNWN. R..LL.RK.. E..TY

Desu-DRB*034CP .....MEYST S.....N... ...Y.D.Y..N ...F...... V..Y...... ...E...WN. R.EIL.EK.. K..TY

Desu-DRB*037CP .....MESL. S.....N... ...F.E.Y..N ...VM..... V..Y...... ..K..KNWN. R..LL.RK.. E..TY

Desu-DRB*041CP .....MEYV. H.....N... ...Y.Y.DI.N ....L..... V......... ........N. R..FL.DL.. R..TY

Desu-DRB*043CP ..D..ML... ......N... ..........N ...F...... V..Y...... .......WN. ...LL..R.. M..TY

Desu-DRB*044CP .....MESL. S.....N... ...F.E.YY.N ....L..... V..Y...... ..K..KNWN. R..LL.RK.. E..TY

Desu-DRB*046CP .....LV.D. S.....N... ...Y.L.Y..N ...FA..... V..Y...... .......WN. ...LL.RR.. K..TY

Desu-DRB*051CP .....MEYST S.....N... ...Y.D.Y..N ...V...... V..Y...... ...E...WN. R..LL.EK.. K..TY

Desu-DRB*052CP .....MEHV. Y.....N... ...Y.E..V.N ...F...... V..Y...... .....K.LN. ...LL.DR.. M..TY

Desu-DRB*053CP .....L.... L.....N... ...Y.V.Y.SN ...FA..... V..Y...... .......WN. ...LL.RR.. E..TL

Desu-DRB*056_P .....LV.D. S.....N... ...Y.L.Y..N ...F...... V..Y...... .......YN. R..LL..R.. K..TY

Desu-DRB*057CP .....MESL. S.....N... ...F.E.Y..N .......... V......... ..K..KNWN. R..LL.RK.. E..TY

Desu-DRB*058CP .....MESL. S.....N... ...F.Q.Y..N G......... .......... ..T..K.YN. ..E.I.DL.. ....-

Desu-DRB*062CP .....MESL. S.....N... ...F.E.Y..N ...F...... V..Y...... ..K..KNWN. R..LL.RK.. E..TY

Desu-DRB*064CP .....LFH.. ......N... ...Y.E.YI.N ...FA..... V......... ..K..KNWN. R..LL.DR.. S..TY

Desu-DRB*068CP .....MEHV. Y.....N... ...Y.E..V.N ...F...... V..Y...S.. .....K.LN. ...LL.DR.. M..TY

Desu-DRB*069CP .....MESL. S.....N... ...F.E.Y..N .......... V..Y...... ..K..KNWN. R..LL.RK.. E..TY

Desu-DRB*073_P .....L.... L.....N... ...Y.V.Y.SN ...FA..... V..Y...... .......WN. ...LL.RR.. E..TY

Desu-DRB*074CP .....LEHV. F.....N... ...F.V.DI.N ...V...... V......S.. ........N. R..FL.E... G..K-

Desu-DRB*087CP .....LV.D. S.....N... ...Y.L.H..N G..F...... V..Y...... .......WN. ...LL..R.. K..TY

Desu-DRB*091CP .....MESL. S.....N... ...F.Q.Y..N G......... .......... ..T..K.YN. ..E.I.DL.. ...TF

Desu-DRB*098CP .....MESL. S.....N... .....Q.YI.N .......... V......... ..LS....N. R.E.I.DL.. G..TF

Desu-DRB*106CP .....MEYV. H.....N... ...Y.Y.DI.N ....L..... V......... .......YN. R.EIL.EK.. K..TY

Desu-DRB*108CP .....LEHV. F.....N... ...F.V.DI.N ...V...... V......... ........N. R.EIL.E... G..K-

Desu-DRB*115_P .......... G.....N... S.........N .......... V......... .....K.WN. ..E....... ....-

Desu-DRB*117_P .....LKYV. H.....N... ...Y.Y.DI.N ...F...... V..Y...... .......WN. ...LL.DS.. ...TY

Desu-DRB*119_P ..D..ML... ......N... ..........N ...F...... .......... .......WN. ..E.I..... ...TY

Desu-DRB*120_P .......... G.....N... S.........N .......... V......... .....K.WN. ..EIL..... ....-

Desu-DRB*123_P .....MEYV. H.....N... ...F.Y.DI.N ....L..... V......... .......YN. R.EIL.R... E..K-

Desu-DRB*124_P .....MEYV. H.....N... ...F.Y.DI.N ....L..... V..Y...... ........N. R.EIL.EK.. K..TY

**Figure S2:** Alignment of amino acid sequences of *D. sublineatus* MHC class II DRB alleles detected by cloning/Sanger sequencing (C) and/or 454 pyrosequencing (P). Dots represent sequence identity with the allele Desu-DRB*01. Numeration is according to the human DR1 molecule and asterisks indicate the human antigen binding sites (ABS) defined by Brown et al [42]. ConsSites mark conserved amino acids across all alleles.

**Figure S3**

**Figure S3: Allele frequencies in individuals genotyped by cloning/Sanger sequencing and 454 pyrosequencing.** The number of alleles detected by cloning/Sanger and 454 pyrosequencing are indicated by blue bars. Red bars indicate the additional alleles detected only by 454 pyrosequencing.

**Figure S4**

**Figure S4:** Comparison of levels of individual MHC class II DRB diversity obtained by conventional cloning/Sanger sequencing and next-generation 454 pyrosequencing. The number of alleles detected by cloning/Sanger and 454 pyrosequencing are indicated by blue bars. Red bars indicate the additional alleles only detected by 454 pyrosequencing. Dotted bars indicate 454 low efficiency alleles.

**Figure S5:**

**
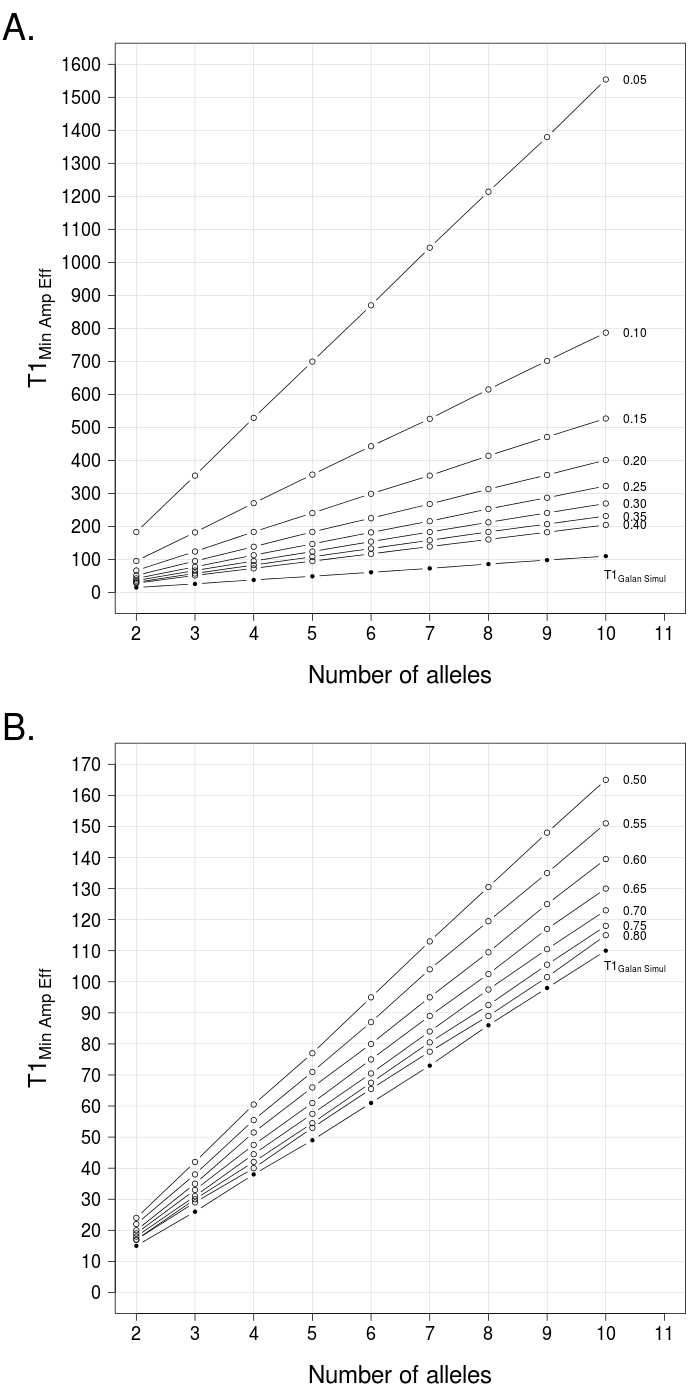
**

**Figure S5: Predicted minimum number of reads (T1_Min Amp Eff_) required to determine a complete genotype for at least two reads per allele (99.9% confidence level).** Lines provide T1 values for given levels of minimum amplification efficiency (indicated at the end of each line) and different numbers of alleles (x-axis). The line in bold indicates T1_Min Amp Eff_ when all alleles have an amplification efficiency of one and therefore correspond to T1_Galan Simul_. The figure has been split into two plots for graphical purpose only. T1_Min Amp Eff_ is given here for a genotyping coverage requiring the presence of at least two reads per allele. For exact values and more details about amplification efficiencies, refer to Additional data file 2 Table S3.

**Figure S6:**


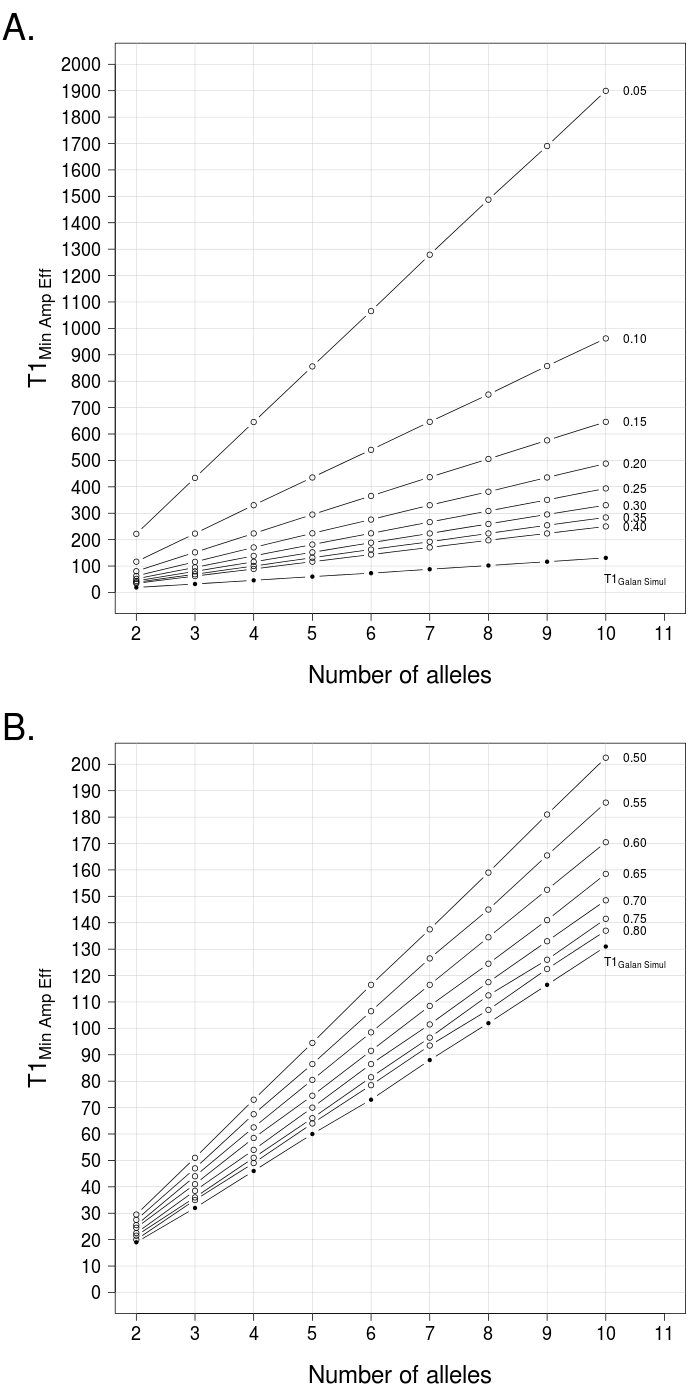


**Figure S6: Predicted minimum number of reads (T1_Min Amp Eff_) required to determine a complete genotype for at least three reads per allele (99.9% confidence level).** T1_Min Amp Eff_ is given here for a genotyping coverage requiring the presence of at least three reads per allele. For exact values and more details about amplification efficiencies, refer to Additional data file 2 Table S4.

**Additional References**

Brown JH, Jardetzky TS, Gorga JC, Stern LJ: **Three-dimensional structure of the human class II histocompatibility antigen HLA-DR1.** *Nature* 1993, **364:**33-39.
