# Supplementary material for: MHC genotyping of non-model organisms using next-generation sequencing: a new methodology to deal with artefacts and allelic dropout
Source: BMC Genomics. 2013 Aug 9;14:542. doi: 10.1186/1471-2164-14-542 (PMC3750822; doi:10.1186/1471-2164-14-542)
Supplement: Additional file 2: Table S1 — Shows the MHC-DRB diversity in D. sublineatus detected by cloning/Sanger sequencing and 454 pyrosequencing using the identical individuals. Table S2 shows a list of the standardised allele amplification efficiencies. Table S3 shows the predicted minimum number of reads necessary to obtain at least two reads per allele based on minimum amplification efficiency. Table S4 shows the predicted minimum number of reads necessary to obtain at least three reads per allele based on minimum amplification efficiency. [file 1471-2164-14-542-S2.docx]

**Additional data file 2 contains supplementary Tables.**

**Table S1:** MHC-DRB diversity in *D. sublineatus* detected by cloning/Sanger sequencing and 454 pyrosequencing using the identical individuals (n= 36)*.* The number of detected alleles, inferred minimum number of DRB loci, as well as variable/total number of positions and the mean number of differences between alleles (± standard error) are displayed.

|  |  |  | Nucleotide sequence | | Amino acid sequence | |
| --- | --- | --- | --- | --- | --- | --- |
| Approach | DRB alleles | DRB loci | variable positions | Ø diff. | variable positions | Ø diff. |
| Cloning/Sanger sequencing | 52/49 | ≥ 4 | 90/228 (39.5%) | 29.0 ± 2.9 | 36/76 (47.7%) | 16.4 ± 2.5 |
|  |  |  |  |  |  |  |
| 454 pyro-sequencing | 64/57 | ≥ 5 | 93/228 (40.8%) | 29.3 ± 2.9 | 37/76 (48.7%) | 16.3 ± 2.5 |
|  |  |  |  |  |  |  |

**Table S2: Standardized allele amplification efficiencies.** For each nucleotide allele, the amplification efficiency was estimated by maximum likelihood and scaled using the first allele as a reference. The number of individuals for which alleles have been identified are also provided.

| **Allele #** | **Amplification efficiency** | **N individuals** |
| --- | --- | --- |
| 001a_CP | 1.00 | 4 |
| 002_CP | 1.13 | 7 |
| 003_CP | 1.53 | 4 |
| 004a_CP | 0.81 | 1 |
| 005_CP | 0.69 | 1 |
| 006_CP | 0.84 | 13 |
| 008_CP | 0.84 | 1 |
| 010a_CP | 0.85 | 2 |
| 010b_CP | 1.03 | 2 |
| 011_CP | 0.92 | 12 |
| 012a_CP | 0.89 | 3 |
| 013_CP | 0.71 | 3 |
| 014a_CP | 0.38 | 3 |
| 014b_CP | 1.28 | 6 |
| 015_CP | 0.64 | 2 |
| 016_CP | 0.92 | 7 |
| 017a_CP | 0.50 | 7 |
| 017b_CP | 0.53 | 4 |
| 018_CP | 0.51 | 1 |
| 019_CP | 0.69 | 10 |
| 020b_CP | 1.00 | 3 |
| 022a_CP | 1.04 | 3 |
| 025_CP | 0.44 | 3 |
| 026_CP | 0.43 | 3 |
| 027_CP | 0.89 | 7 |
| 028_P | 0.19 | 1 |
| 029_CP | 0.87 | 2 |
| 031b_CP | 0.85 | 3 |
| 032_CP | 0.57 | 2 |
| 033_CP | 0.61 | 4 |
| 034_CP | 0.66 | 2 |
| 037_CP | 0.44 | 1 |
| 041_CP | 0.37 | 2 |
| 043_CP | 0.57 | 1 |
| 044b_CP | 0.35 | 4 |
| 046a_CP | 0.46 | 1 |
| 046b_CP | 0.56 | 3 |
| 051_CP | 0.71 | 3 |
| 052_CP | 1.18 | 1 |
| 056_P | 0.58 | 1 |
| 057a_CP | 0.78 | 9 |
| 058a_CP | 1.50 | 4 |
| 058b_P | 0.69 | 1 |
| 062a_CP | 0.36 | 4 |
| 062b_CP | 0.75 | 4 |
| 064_CP | 0.50 | 4 |
| 068_CP | 1.01 | 4 |
| 069_CP | 0.97 | 4 |
| 073_P | 0.76 | 1 |
| 074_CP | 0.30 | 1 |
| 087_CP | 0.72 | 2 |
| 091_CP | 2.40 | 4 |
| 098_CP | 0.75 | 1 |
| 106_CP | 0.51 | 1 |
| 108_CP | 0.40 | 1 |
| 115_P | 1.34 | 1 |
| 117_P | 1.35 | 1 |
| 119_P | 0.52 | 2 |
| 120_P | 0.89 | 1 |
| 123_P | 0.97 | 1 |
| 124_P | 0.74 | 1 |
|  |  |  |

**Table S3: Predicted minimum number of reads (T1_Min Amp Eff_) required to determine a complete genotype for at least two reads per allele (99.9% confidence level).** T1_Min Amp Eff_ is given here for a genotyping coverage requiring the presence of at least two reads per allele, according to levels of minimum amplification efficiency (in rows) and different numbers of alleles (in columns).

| Amplification efficiency of the  least efficient allele | Number of alleles | | | | | | | | |
| --- | --- | --- | --- | --- | --- | --- | --- | --- | --- |
|  | 2 | 3 | 4 | 5 | 6 | 7 | 8 | 9 | 10 |
| 0.99 | 15 | 26 | 38 | 49 | 61 | 73 | 86 | 98 | 110 |
| 0.98 | 15 | 27 | 38 | 50 | 61 | 73 | 86 | 99 | 110 |
| 0.97 | 15 | 26 | 38 | 50 | 61 | 73 | 86 | 99 | 110 |
| 0.96 | 15 | 27 | 38 | 50 | 61 | 73 | 86 | 99 | 111 |
| 0.95 | 15 | 27 | 38 | 50 | 62 | 74 | 86 | 98 | 111 |
| 0.94 | 15 | 27 | 38 | 50 | 62 | 74 | 86 | 98 | 110 |
| 0.93 | 16 | 27 | 38 | 50 | 61 | 74 | 86 | 98 | 110 |
| 0.92 | 16 | 27 | 38 | 50 | 62 | 74 | 86 | 98 | 110 |
| 0.91 | 16 | 27 | 38 | 50 | 62 | 74 | 86 | 98 | 111 |
| 0.90 | 16 | 27 | 38 | 50 | 62 | 74 | 86 | 99 | 111 |
| 0.89 | 16 | 27 | 39 | 50 | 62 | 74 | 86 | 100 | 111 |
| 0.88 | 16 | 27 | 39 | 50 | 62 | 74 | 87 | 100 | 111 |
| 0.87 | 16 | 27 | 39 | 51 | 63 | 75 | 87 | 100 | 112 |
| 0.86 | 16 | 27 | 39 | 51 | 63 | 76 | 87 | 100 | 112 |
| 0.85 | 16 | 28 | 39 | 51 | 63 | 76 | 88 | 101 | 112 |
| 0.84 | 16 | 28 | 39 | 51 | 63 | 76 | 87 | 101 | 112 |
| 0.83 | 16 | 28 | 40 | 51 | 64 | 77 | 88 | 101 | 113 |
| 0.82 | 16 | 28 | 40 | 52 | 65 | 77 | 89 | 102 | 114 |
| 0.81 | 17 | 28 | 40 | 52 | 66 | 77 | 89 | 102 | 114 |
| 0.80 | 17 | 29 | 40 | 53 | 66 | 78 | 89 | 102 | 115 |
| 0.79 | 17 | 29 | 41 | 53 | 66 | 78 | 90 | 103 | 115 |
| 0.78 | 17 | 29 | 41 | 53 | 67 | 79 | 91 | 104 | 116 |
| 0.77 | 17 | 29 | 41 | 54 | 67 | 80 | 92 | 104 | 116 |
| 0.76 | 17 | 29 | 42 | 54 | 68 | 80 | 93 | 105 | 117 |
| 0.75 | 17 | 30 | 42 | 55 | 68 | 81 | 93 | 106 | 118 |
| 0.74 | 18 | 30 | 43 | 56 | 69 | 81 | 94 | 107 | 119 |
| 0.73 | 18 | 30 | 43 | 56 | 69 | 82 | 95 | 108 | 120 |
| 0.72 | 18 | 31 | 44 | 57 | 70 | 83 | 96 | 109 | 121 |
| 0.71 | 18 | 31 | 45 | 58 | 70 | 84 | 97 | 109 | 122 |
| 0.70 | 18 | 31 | 45 | 58 | 71 | 84 | 98 | 111 | 123 |
| 0.69 | 19 | 32 | 46 | 59 | 72 | 84 | 99 | 112 | 124 |
| 0.68 | 19 | 32 | 46 | 60 | 73 | 86 | 100 | 113 | 126 |
| 0.67 | 19 | 33 | 47 | 60 | 74 | 86 | 101 | 114 | 127 |
| 0.66 | 19 | 33 | 48 | 61 | 75 | 87 | 102 | 114 | 128 |
| 0.65 | 19 | 33 | 48 | 61 | 75 | 89 | 103 | 117 | 130 |
| 0.64 | 20 | 34 | 49 | 62 | 76 | 90 | 105 | 118 | 131 |
| 0.63 | 20 | 34 | 50 | 63 | 77 | 91 | 106 | 119 | 134 |
| 0.62 | 20 | 34 | 51 | 63 | 78 | 92 | 108 | 121 | 135 |
| 0.61 | 20 | 35 | 51 | 64 | 79 | 94 | 109 | 123 | 138 |
| 0.60 | 20 | 35 | 52 | 66 | 80 | 95 | 110 | 125 | 140 |
| 0.59 | 21 | 36 | 52 | 67 | 82 | 96 | 112 | 126 | 142 |
| 0.58 | 21 | 37 | 53 | 67 | 83 | 98 | 115 | 129 | 145 |
| 0.57 | 21 | 37 | 54 | 69 | 84 | 100 | 116 | 131 | 146 |
| 0.56 | 22 | 38 | 55 | 70 | 86 | 101 | 118 | 132 | 148 |
| 0.55 | 22 | 38 | 56 | 71 | 87 | 104 | 120 | 135 | 151 |
| 0.54 | 22 | 39 | 57 | 72 | 88 | 105 | 122 | 137 | 154 |
| 0.53 | 22 | 40 | 57 | 73 | 90 | 107 | 124 | 139 | 156 |
| 0.52 | 23 | 40 | 58 | 75 | 92 | 109 | 125 | 143 | 159 |
| 0.51 | 23 | 41 | 59 | 76 | 93 | 111 | 129 | 145 | 162 |
| 0.50 | 24 | 42 | 61 | 77 | 95 | 113 | 131 | 148 | 165 |
| 0.49 | 24 | 43 | 62 | 79 | 96 | 115 | 134 | 150 | 168 |
| 0.48 | 24 | 44 | 63 | 80 | 99 | 117 | 136 | 154 | 172 |
| 0.47 | 25 | 45 | 64 | 82 | 101 | 119 | 139 | 158 | 175 |
| 0.46 | 25 | 46 | 65 | 83 | 102 | 122 | 142 | 161 | 179 |
| 0.45 | 26 | 47 | 66 | 85 | 104 | 125 | 145 | 164 | 183 |
| 0.44 | 26 | 48 | 67 | 87 | 107 | 128 | 148 | 167 | 188 |
| 0.43 | 26 | 48 | 68 | 88 | 110 | 130 | 151 | 171 | 191 |
| 0.42 | 27 | 50 | 69 | 91 | 112 | 133 | 154 | 174 | 196 |
| 0.41 | 27 | 51 | 71 | 93 | 115 | 136 | 158 | 179 | 201 |
| 0.40 | 28 | 52 | 73 | 95 | 117 | 139 | 161 | 183 | 205 |
| 0.39 | 29 | 53 | 75 | 97 | 120 | 143 | 166 | 188 | 211 |
| 0.38 | 29 | 54 | 76 | 100 | 122 | 146 | 169 | 193 | 216 |
| 0.37 | 30 | 55 | 78 | 101 | 126 | 149 | 175 | 197 | 221 |
| 0.36 | 31 | 56 | 80 | 105 | 129 | 154 | 179 | 203 | 226 |
| 0.35 | 31 | 58 | 83 | 108 | 133 | 158 | 184 | 207 | 232 |
| 0.34 | 32 | 59 | 84 | 110 | 136 | 162 | 188 | 213 | 240 |
| 0.33 | 33 | 61 | 87 | 114 | 140 | 167 | 194 | 220 | 247 |
| 0.32 | 34 | 63 | 89 | 117 | 144 | 171 | 200 | 226 | 254 |
| 0.31 | 35 | 64 | 92 | 120 | 148 | 177 | 206 | 233 | 262 |
| 0.30 | 36 | 67 | 95 | 124 | 154 | 183 | 213 | 241 | 270 |
| 0.29 | 37 | 69 | 98 | 128 | 158 | 188 | 220 | 249 | 280 |
| 0.28 | 38 | 71 | 101 | 132 | 164 | 194 | 227 | 258 | 290 |
| 0.27 | 39 | 73 | 104 | 137 | 169 | 202 | 235 | 266 | 299 |
| 0.26 | 41 | 76 | 108 | 142 | 177 | 209 | 242 | 276 | 311 |
| 0.25 | 43 | 78 | 113 | 147 | 182 | 216 | 253 | 287 | 323 |
| 0.24 | 44 | 81 | 117 | 154 | 190 | 226 | 262 | 299 | 336 |
| 0.23 | 46 | 84 | 122 | 159 | 199 | 235 | 275 | 312 | 348 |
| 0.22 | 48 | 88 | 127 | 167 | 207 | 247 | 285 | 324 | 363 |
| 0.21 | 49 | 91 | 132 | 176 | 216 | 257 | 298 | 339 | 380 |
| 0.20 | 52 | 95 | 139 | 184 | 225 | 268 | 313 | 356 | 401 |
| 0.19 | 54 | 100 | 146 | 192 | 236 | 284 | 328 | 376 | 421 |
| 0.18 | 57 | 105 | 154 | 203 | 250 | 299 | 347 | 397 | 443 |
| 0.17 | 59 | 110 | 164 | 215 | 265 | 315 | 365 | 418 | 468 |
| 0.16 | 62 | 117 | 173 | 225 | 280 | 334 | 389 | 442 | 496 |
| 0.15 | 67 | 124 | 184 | 241 | 299 | 354 | 414 | 471 | 527 |
| 0.14 | 70 | 133 | 196 | 257 | 319 | 380 | 443 | 504 | 567 |
| 0.13 | 75 | 142 | 211 | 278 | 342 | 409 | 477 | 542 | 607 |
| 0.12 | 81 | 153 | 227 | 299 | 370 | 441 | 516 | 586 | 659 |
| 0.11 | 88 | 167 | 247 | 325 | 404 | 481 | 561 | 638 | 715 |
| 0.10 | 96 | 182 | 271 | 357 | 443 | 526 | 615 | 702 | 787 |
| 0.09 | 105 | 202 | 299 | 393 | 490 | 586 | 681 | 776 | 870 |
| 0.08 | 118 | 226 | 335 | 444 | 551 | 659 | 764 | 871 | 979 |
| 0.07 | 133 | 256 | 381 | 504 | 625 | 751 | 872 | 991 | 1115 |
| 0.06 | 154 | 298 | 443 | 586 | 726 | 870 | 1015 | 1155 | 1304 |
| 0.05 | 183 | 354 | 529 | 700 | 870 | 1045 | 1214 | 1380 | 1554 |
| 0.04 | 226 | 442 | 660 | 870 | 1087 | 1298 | 1513 | 1725 | 1935 |
| 0.03 | 299 | 586 | 870 | 1159 | 1441 | 1715 | 2006 | 2288 | 2568 |
| 0.02 | 441 | 874 | 1299 | 1715 | 2145 | 2572 | 2989 | 3410 | 3832 |
| 0.01 | 871 | 1731 | 2570 | 3409 | 4263 | 5094 | 5936 | 6773 | 7585 |

**Table S4: Predicted minimum number of reads (T1_Min Amp Eff_) required to determine a complete genotype for at least three reads per allele (99.9% confidence level).** T1_Min Amp Eff_ is given here for a genotyping coverage requiring the presence of at least three reads per allele, according to levels of minimum amplification efficiency (in rows) and different numbers of alleles (in columns).

| Amplification efficiency of the  least efficient allele | Number of alleles | | | | | | | | |
| --- | --- | --- | --- | --- | --- | --- | --- | --- | --- |
|  | 2 | 3 | 4 | 5 | 6 | 7 | 8 | 9 | 10 |
| 1.00 | 19 | 32 | 46 | 60 | 73 | 88 | 102 | 117 | 131 |
| 0.99 | 19 | 32 | 46 | 60 | 74 | 88 | 102 | 118 | 131 |
| 0.98 | 19 | 32 | 46 | 59 | 74 | 88 | 102 | 118 | 131 |
| 0.97 | 19 | 32 | 46 | 59 | 74 | 88 | 102 | 118 | 131 |
| 0.96 | 19 | 32 | 46 | 60 | 74 | 88 | 102 | 118 | 131 |
| 0.95 | 19 | 32 | 46 | 60 | 75 | 88 | 102 | 118 | 131 |
| 0.94 | 19 | 32 | 46 | 60 | 75 | 88 | 102 | 118 | 132 |
| 0.93 | 19 | 32 | 46 | 60 | 75 | 88 | 102 | 118 | 132 |
| 0.92 | 19 | 33 | 46 | 60 | 75 | 88 | 103 | 118 | 132 |
| 0.91 | 19 | 33 | 46 | 60 | 75 | 88 | 102 | 118 | 132 |
| 0.90 | 19 | 33 | 46 | 60 | 75 | 88 | 103 | 119 | 132 |
| 0.89 | 19 | 33 | 47 | 61 | 75 | 89 | 103 | 119 | 132 |
| 0.88 | 19 | 33 | 47 | 61 | 76 | 90 | 104 | 119 | 133 |
| 0.87 | 20 | 33 | 47 | 61 | 76 | 91 | 104 | 119 | 133 |
| 0.86 | 20 | 34 | 47 | 61 | 76 | 90 | 104 | 119 | 134 |
| 0.85 | 20 | 34 | 47 | 62 | 77 | 91 | 105 | 120 | 133 |
| 0.84 | 20 | 34 | 48 | 62 | 77 | 92 | 105 | 121 | 134 |
| 0.83 | 20 | 34 | 48 | 62 | 78 | 92 | 106 | 121 | 135 |
| 0.82 | 20 | 34 | 48 | 63 | 78 | 92 | 106 | 122 | 135 |
| 0.81 | 20 | 35 | 49 | 63 | 79 | 93 | 107 | 122 | 136 |
| 0.80 | 20 | 35 | 49 | 64 | 79 | 94 | 107 | 123 | 137 |
| 0.79 | 21 | 35 | 49 | 64 | 79 | 94 | 108 | 124 | 138 |
| 0.78 | 21 | 35 | 50 | 65 | 80 | 95 | 109 | 124 | 138 |
| 0.77 | 21 | 36 | 50 | 65 | 81 | 96 | 110 | 126 | 140 |
| 0.76 | 21 | 36 | 51 | 66 | 81 | 96 | 112 | 126 | 141 |
| 0.75 | 22 | 36 | 51 | 66 | 82 | 97 | 113 | 126 | 142 |
| 0.74 | 23 | 37 | 52 | 67 | 83 | 98 | 113 | 128 | 144 |
| 0.73 | 23 | 37 | 52 | 68 | 84 | 99 | 114 | 129 | 145 |
| 0.72 | 23 | 38 | 53 | 68 | 85 | 100 | 116 | 131 | 146 |
| 0.71 | 23 | 38 | 54 | 69 | 86 | 101 | 117 | 132 | 148 |
| 0.70 | 23 | 39 | 54 | 70 | 87 | 102 | 118 | 133 | 149 |
| 0.69 | 24 | 40 | 55 | 71 | 88 | 104 | 120 | 134 | 151 |
| 0.68 | 24 | 40 | 56 | 72 | 89 | 105 | 121 | 136 | 153 |
| 0.67 | 24 | 41 | 57 | 72 | 90 | 106 | 122 | 138 | 155 |
| 0.66 | 25 | 41 | 58 | 73 | 91 | 108 | 125 | 139 | 157 |
| 0.65 | 25 | 41 | 59 | 75 | 92 | 109 | 125 | 141 | 159 |
| 0.64 | 25 | 41 | 60 | 77 | 94 | 111 | 128 | 144 | 161 |
| 0.63 | 25 | 42 | 61 | 78 | 95 | 112 | 129 | 146 | 164 |
| 0.62 | 26 | 42 | 61 | 79 | 96 | 113 | 131 | 149 | 165 |
| 0.61 | 26 | 43 | 62 | 80 | 97 | 115 | 133 | 151 | 169 |
| 0.60 | 26 | 44 | 63 | 81 | 99 | 117 | 135 | 153 | 171 |
| 0.59 | 27 | 44 | 64 | 82 | 101 | 119 | 137 | 156 | 174 |
| 0.58 | 27 | 45 | 65 | 84 | 102 | 121 | 138 | 158 | 176 |
| 0.57 | 27 | 46 | 66 | 84 | 104 | 122 | 140 | 160 | 179 |
| 0.56 | 28 | 46 | 67 | 86 | 105 | 125 | 142 | 164 | 183 |
| 0.55 | 28 | 47 | 68 | 87 | 107 | 127 | 145 | 166 | 186 |
| 0.54 | 28 | 48 | 69 | 89 | 109 | 129 | 147 | 169 | 188 |
| 0.53 | 29 | 49 | 70 | 91 | 111 | 131 | 151 | 172 | 192 |
| 0.52 | 29 | 49 | 72 | 92 | 112 | 133 | 153 | 175 | 195 |
| 0.51 | 30 | 50 | 72 | 94 | 115 | 136 | 156 | 178 | 199 |
| 0.50 | 30 | 51 | 73 | 95 | 117 | 138 | 159 | 181 | 203 |
| 0.49 | 30 | 52 | 74 | 97 | 119 | 141 | 162 | 184 | 206 |
| 0.48 | 31 | 53 | 75 | 99 | 122 | 144 | 165 | 188 | 211 |
| 0.47 | 31 | 54 | 77 | 101 | 124 | 147 | 168 | 191 | 214 |
| 0.46 | 32 | 55 | 78 | 103 | 126 | 149 | 173 | 195 | 220 |
| 0.45 | 32 | 56 | 80 | 105 | 129 | 152 | 175 | 200 | 225 |
| 0.44 | 33 | 57 | 82 | 107 | 131 | 155 | 180 | 203 | 229 |
| 0.43 | 34 | 58 | 83 | 110 | 134 | 159 | 185 | 208 | 234 |
| 0.42 | 34 | 59 | 84 | 112 | 137 | 163 | 188 | 213 | 240 |
| 0.41 | 35 | 60 | 87 | 115 | 141 | 167 | 193 | 218 | 244 |
| 0.40 | 35 | 62 | 89 | 117 | 144 | 171 | 198 | 224 | 250 |
| 0.39 | 36 | 63 | 91 | 120 | 147 | 175 | 203 | 230 | 256 |
| 0.38 | 37 | 65 | 93 | 122 | 151 | 179 | 207 | 235 | 263 |
| 0.37 | 38 | 66 | 96 | 125 | 155 | 184 | 213 | 242 | 269 |
| 0.36 | 39 | 68 | 98 | 130 | 159 | 188 | 219 | 248 | 277 |
| 0.35 | 39 | 69 | 100 | 132 | 163 | 192 | 224 | 255 | 284 |
| 0.34 | 40 | 72 | 103 | 136 | 167 | 198 | 230 | 263 | 292 |
| 0.33 | 42 | 73 | 107 | 140 | 173 | 203 | 237 | 269 | 301 |
| 0.32 | 43 | 76 | 109 | 143 | 177 | 211 | 244 | 278 | 310 |
| 0.31 | 44 | 77 | 112 | 148 | 183 | 217 | 252 | 286 | 319 |
| 0.30 | 44 | 80 | 116 | 152 | 189 | 224 | 260 | 296 | 331 |
| 0.29 | 46 | 83 | 120 | 156 | 195 | 231 | 269 | 305 | 340 |
| 0.28 | 48 | 85 | 124 | 162 | 200 | 239 | 277 | 315 | 354 |
| 0.27 | 49 | 88 | 128 | 168 | 209 | 247 | 288 | 326 | 365 |
| 0.26 | 50 | 91 | 133 | 175 | 216 | 257 | 299 | 339 | 379 |
| 0.25 | 52 | 95 | 139 | 182 | 224 | 267 | 309 | 351 | 394 |
| 0.24 | 54 | 98 | 143 | 189 | 233 | 278 | 321 | 366 | 409 |
| 0.23 | 56 | 102 | 150 | 196 | 241 | 288 | 335 | 382 | 427 |
| 0.22 | 58 | 106 | 156 | 205 | 251 | 301 | 350 | 399 | 445 |
| 0.21 | 60 | 111 | 163 | 213 | 263 | 315 | 365 | 417 | 465 |
| 0.20 | 63 | 116 | 171 | 224 | 276 | 331 | 382 | 436 | 488 |
| 0.19 | 66 | 122 | 180 | 234 | 290 | 347 | 402 | 459 | 513 |
| 0.18 | 69 | 128 | 188 | 247 | 305 | 366 | 425 | 483 | 541 |
| 0.17 | 72 | 135 | 198 | 260 | 323 | 388 | 449 | 511 | 572 |
| 0.16 | 77 | 143 | 211 | 277 | 342 | 410 | 476 | 542 | 608 |
| 0.15 | 81 | 152 | 224 | 295 | 366 | 437 | 506 | 576 | 646 |
| 0.14 | 86 | 162 | 239 | 315 | 390 | 467 | 541 | 617 | 691 |
| 0.13 | 92 | 173 | 257 | 338 | 420 | 502 | 583 | 663 | 745 |
| 0.12 | 99 | 188 | 278 | 365 | 453 | 541 | 630 | 718 | 806 |
| 0.11 | 107 | 203 | 302 | 398 | 492 | 589 | 685 | 783 | 875 |
| 0.10 | 117 | 223 | 331 | 436 | 540 | 646 | 750 | 858 | 962 |
| 0.09 | 128 | 247 | 365 | 483 | 600 | 718 | 835 | 949 | 1065 |
| 0.08 | 144 | 278 | 410 | 543 | 672 | 803 | 938 | 1068 | 1199 |
| 0.07 | 163 | 314 | 466 | 619 | 768 | 921 | 1066 | 1217 | 1367 |
| 0.06 | 188 | 365 | 540 | 719 | 893 | 1070 | 1244 | 1421 | 1586 |
| 0.05 | 222 | 434 | 646 | 856 | 1066 | 1279 | 1488 | 1691 | 1900 |
| 0.04 | 277 | 543 | 806 | 1065 | 1331 | 1589 | 1848 | 2111 | 2373 |
| 0.03 | 364 | 719 | 1067 | 1417 | 1761 | 2108 | 2453 | 2807 | 3143 |
| 0.02 | 542 | 1069 | 1589 | 2117 | 2634 | 3160 | 3665 | 4176 | 4705 |
| 0.01 | 1069 | 2113 | 3149 | 4175 | 5234 | 6260 | 7310 | 8305 | 9352 |
