# Supplementary material for: MHC genotyping of non-model organisms using next-generation sequencing: a new methodology to deal with artefacts and allelic dropout
Source: BMC Genomics. 2013 Aug 9;14:542. doi: 10.1186/1471-2164-14-542 (PMC3750822; doi:10.1186/1471-2164-14-542)
Supplement: Additional file 3: Text S1 — Describes possible adjustments of the reads filtering pathway (Figure 1) and our allele and artefact identification workflow (Figure 2) to different needs in forthcoming MHC studies. [file 1471-2164-14-542-S3.docx]

**Possible adjustments of the reads filtering pathway (Figure 1) and our allele and artefact identification workflow (Figure 2)** **to different needs in forthcoming MHC studies**

We proposed a very conservative initial 454 data quality check and reads filtering step (Figure 1). Any error in the read will cause its elimination from the data set, which means that the read is not influencing allele frequencies. This filtering facilitates the analysis by excluding indels and many mistakes yielded by pyrosequencing. However, before applying such filtering, it is crucial that the scientist checks for systematics errors that might arise during base calling and initial 454 standard quality filtering steps. For that we have re-run the signal processing after switching off as many filters as the 454 software allows. The results did not reveal any specific changes in our results or biases towards read filtering or trimming, thereby demonstrating that our 454 sequencing run yielded a high number of unbiased good quality reads. However, we have experienced examples of systematic biases in other studies (data not shown). For example, reads possessing internal regions with off-peak signal intensity (common in homopolymeric stretches) might end up trimmed or discarded automatically by the ValleyFilterTrimBack filter of the 454 software. This could certainly create a bias against alleles presenting more or longer homopolymeric regions. In cases where the filtering process induces biases, we recommend turning the ValleyFilterTrimBack off in order to recover most sequences and then adapt our initial filtering steps to not discard such sequences due to lower quality scores. Also, artificial indels are often produced during base calling, and can be systematically repeated among reads. This could again be biased towards some specific alleles. The latter problem can be addressed by changes in the initial filtering steps as well as in the allele and artefact identification workflow, but the exact nature of modifications depends on the type of bias observed.

Depending on the species-specific MHC complexity, slight adjustments in our allele and artefact identification workflow (Figure 2) might facilitate the analyses. First, in species with recent gene duplications leading to many closely related ‘putative alleles’, one can change the ‘1-2bp diff’ and ‘>2bp diff’ intra-amplicon evaluation categories into ‘1bp diff’ and ‘>1bp diff’. In our case, we have found a much higher amount of artefacts with one base pair difference to a ‘putative allele’ than two base pairs. True alleles were in general very different from each other (average = 29.3 bp ± 2.9), which allowed us to consider most of the variants with 1-2 bp differences to an allele as artefacts. Second, studies in species or populations with limited amounts of alleles, where all of them are believed to be previously known, probably do not need to include amplicon replicates for each individual. In this case, step II of our workflow (Figure 2) could be modified, such that instead of checking for presence of a variant in the same individual replicate, tests would be done among individuals, since many individuals would tend to share the same alleles. However, we would recommend a partial amount of replicates to be run in the first sequencing procedure in order to assess the overall genotyping reliability and to allow enough time and financial support in case more replicates are required.
