# Supplementary material for: MHC genotyping of non-model organisms using next-generation sequencing: a new methodology to deal with artefacts and allelic dropout
Source: BMC Genomics. 2013 Aug 9;14:542. doi: 10.1186/1471-2164-14-542 (PMC3750822; doi:10.1186/1471-2164-14-542)
Supplement: Additional file 4 — The R-Codes used to estimate the allele’s amplification efficiency and the different T1 thresholds. [file 1471-2164-14-542-S4.pdf]

Additional data file 4:
R codes

Sommer S, Courtiol A & Mazzoni CJ

The following text details how to implement our analyses using the free statistical software R (1). This document has been created using the R package `knitr` (2). A basic knowledge of R is required to follow the procedure, nonetheless we will try to provide as much details as possible. We also tried to find a good balance between clarity and effectiveness for coding. Both R and its manuals are freely available at http://cran.r-project.org/.

1 Data input

We will start by loading the dataset containing read numbers per amplicon into R. The exact details depend on the structure of your file and on where your file is in your computer, but the principle is to use the function `read.table()`. Just type `?read.table()` in the R terminal to access its documentation.

We load the dataset:

```
matrix.data.temp <- read.table("Matrix_IndivXAlleles.txt", header = T)
```

Because in our data file the two first columns contain information we do not need here, we remove them:

```
matrix.data <- matrix.data.temp[, c(-1, -2)]
```

The object `matrix.data` is a matrix where each row corresponds to an amplicon and each column to an allele. In this matrix, the cell indicates the observed number of reads. Note that pairs of consecutive rows present similar number of reads because they correspond to the two amplicon replicates.

We display the 6 first rows of the dataset:

```
head(matrix.data)
```

```
##   X001a_CP X002_CP X003_CP X004a_CP X005_CP X006_CP X008_CP X010a_CP
## 1        0       0       0        0       0       0       0        0
## 2        0       0       0        0       0       0       0        0
## 3        0       0       0        0       0       0       0        0
## 4        0       0       0        0       0       0       0        0
## 5        0       0       0        0       0     114       0        0
## 6        0       0       0        0       0      77       0        0
##   X010b_CP X011_CP X012a_CP X013_CP X014a_CP X014b_CP X015_CP X016_CP
## 1       66       0        0       0        0        0       0       0
## 2       75       0        0       0        0        0       0       0
## 3        0      45        0       0        0      136       0       0
## 4        0      27        0       0        0       83       0       0
## 5        0       0        0       0        0        0       0       0
## 6        0       0        0       0        0        0       0       0
##   X017a_CP X017b_CP X018_CP X019_CP X020b_CP X022a_CP X025_CP X026_CP
## 1        0       14       0       0        0        0       0       0
## 2        0       52       0       0        0        0       0       0
## 3        0        0       0      50        0        0      32      23
## 4        0        0       0      32        0        0      32      13
## 5       28        0       0       0        0        0       0       0
## 6       39        0       0       0        0        0       0       0
##   X027_CP X028_CP X029_CP X031b_CP X032_CP X033_CP X034_CP X037_CP X041_CP
## 1       0       0       0        0       0       0       0       0       0
## 2       0       0       0        0       0       0       0       0       0
## 3       0       0       0        0       0      43       0       0       0
## 4       0       0       0        0       0      18       0       0       0
## 5       0       0       0        0       0       0       0       0       0
## 6       0       0       0        0       0       0       0       0       0
##   X043_CP X044b_CP X046a_CP X046b_CP X051_CP X052_CP X056_CP X057a_CP
## 1       0        0        0        0      46       0       0        0
## 2       0        0        0        0      63       0       0        0
## 3       0        0        0        0       0       0       0        0
## 4       0        0        0        0       0       0       0        0
## 5       0        0        0        0       0       0       0        0
## 6       0        0        0        0       0       0       0        0
##   X058a_CP X058b_P X062a_CP X062b_CP X064_CP X068_CP X069_CP X073_CP
## 1        0       0        0        0       0      75      31       0
## 2        0       0        0        0       0      77      66       0
## 3        0       0        0        0       0       0       0       0
## 4        0       0        0        0       0       0       0       0
## 5      235       0       39        0       0       0       0       0
## 6      224       0       42        0       0       0       0       0
##   X074_CP X087_CP X091_CP X098_CP X106_CP X108_CP X115_P X117_P X119_P
## 1       0       0       0       0       0       0      0     94      0
## 2       0       0       0       0       0       0      0     85      0
## 3       0       0       0       0       0       0      0      0      0
## 4       0       0       0       0       0       0      0      0      0
## 5       0       0       0       0       0       0      0      0      0
## 6       0       0       0       0       0       0      0      0      0
##   X120_P X123_P X124_P
## 1      0      0      0
## 2      0      0      0
## 3      0      0      0
## 4      0      0      0
## 5      0      0      0
## 6      0      0      0
```

2 Estimation of amplification efficiencies

To estimate the relative amplification efficiencies of the alleles, we designed two functions.

2.1 Defining the function `DensityAmplicon()`

The first function is called `DensityAmplicon()` and is used internally by the second function we will describe afterward.

```
DensityAmplicon <- function(reads, proba, logOutput) {
    # Compute the likelihood or loglikelihood of an amplicon
    #
    # Args:
    #   reads: the observed reads number.
    #   proba: the efficiency values for alleles with non-null read numbers.
    #   logOutput: if TRUE return the logdensity, if FALSE return the density.
    #
    # Returns: the density probability of an amplicon.
    allele.indexes <- which(reads != 0L)
    return(dmultinom(x=reads[allele.indexes], prob=proba[allele.indexes],
      log=logOutput))
}
```

It is a function that will provide the likelihood or loglikelihood of an amplicon, given efficiency values for the alleles. The function `DensityAmplicon()` relies itself on the function `dmultinom()` that provides the density function of the multinomial distribution. This latter function is part of R. Importantly, the function `dmultinom()` requires a set of probabilities summing to one, which is different from amplification efficiencies values that are defined at the level of alleles, irrespectively of the genotype, and that do not necessarily sum to one. Still, because the function `dmultinom()` re-scales all values given as probabilities so that they do sum to one, we can provide directly efficiency values to the `prob` argument of the function.

We can try the function by computing for instance the probability to have observed the number of read associated with our first amplicon assuming equal efficiency between all alleles. Note that we set here the `logOutput` argument to `FALSE` to obtain the result in the probability scale rather than log:

```
nb.alleles <- length(matrix.data[1, ])
efficiencies <- rep(1, nb.alleles)

DensityAmplicon(reads = matrix.data[1, ], proba = efficiencies, logOutput = F)

## [1] 8.802e-26
```

2.2 Defining the function `LoglikData()`

Now we need a function that apply the function `DensityAmplicon()` to all amplicons in order to obtain the loglikelihood of the entire dataset, i.e. the (log) probability of observing the number of reads we have, given the efficiency values. The log version of the likelihood is used because the loglikelihood of the dataset is simply equal to the sum of the individual loglikelihood. Using the non-log scale would imply multiplications and would create numeric problems because individual value can be very small.

```r
LoglikData <- function(proba, data) {
    # Compute the loglikelihood of the dataset
    #
    # Args:
    #   proba: the efficiency values for all alleles.
    #   data: the name of the dataset.
    #
    # Returns: the loglikelihood of the dataset.
    densities <- apply(data, 1,
      function(x) DensityAmplicon(x, proba, logOutput=T))
    return(sum(densities))
}
```

Again, we can try this function on our dataset assuming that all alleles have equal efficiencies.

```r
LoglikData(proba = efficiencies, data = matrix.data)

## [1] -3441
```

2.3 Estimating the relative amplification efficiency of each alleles

So far in the examples, we assumed an efficiency of 1 for all alleles but what we want is actually to estimate the efficiency values from our data. To do that, we will use the function `optim()` from R. Starting from initial values for efficiencies (defined by the `par` argument that we set to equal efficiency values of 1), `optim()` will simulate other efficiency values until the loglikelihood is maximal. Note that the loglikelihood is maximal for the same set of amplification efficiencies that would maximize the likelihood on the non-log scale. Since `optim()` looks for minimum by default, we use the option `fnscale=-1` to search the maximum. Also, `optim()` can use different optimization algorithms (see `?optim()` for details). We chose to use the algorithm refereed as `method="L-BFGS-B"` because it works very well on simulated dataset and it is reasonably fast. This algorithm takes boundaries for parameters (defined by the `upper` and `lower` arguments). We constrained `optim()` to look for efficiency estimates between 0.1 and 6, but if your estimates reach those boundaries, you should modify those limits. Let's also measure meanwhile how long it takes using the function `system.time` (look at the column `elapsed`, the result is expressed in seconds):

```r
system.time(
  efficiencies.relative.temp <- optim(par=efficiencies, fn=LoglikData,
    data=matrix.data, control=list(fnscale=-1), method="L-BFGS-B",
    lower=rep(0.1, nb.alleles), upper=rep(6, nb.alleles)))

##    user  system elapsed
##  16.137   0.396  17.224
```

We display the outcome:

```r
efficiencies.relative.temp
```

```
## $par
##  [1] 1.4900 1.6769 2.2843 1.2121 1.0300 1.2576 1.2488 1.2653 1.5323 1.3761
## [11] 1.3295 1.0624 0.5685 1.9025 0.9463 1.3702 0.7396 0.7923 0.7581 1.0338
## [21] 1.4919 1.5565 0.6572 0.6449 1.3314 0.2815 1.2987 1.2650 0.8513 0.9076
## [31] 0.9876 0.6602 0.5562 0.8516 0.5246 0.6784 0.8364 1.0554 1.7521 0.8620
## [41] 1.1692 2.2392 1.0249 0.5327 1.1127 0.7498 1.5032 1.4480 1.1397 0.4499
## [51] 1.0691 3.5717 1.1237 0.7613 0.5902 1.9996 2.0060 0.7760 1.3247 1.4434
## [61] 1.1021
##
## $value
## [1] -1576
##
## $counts
## function gradient
##       51       51
##
## $convergence
## [1] 0
##
## $message
## [1] "CONVERGENCE: REL_REDUCTION_OF_F <= FACTR*EPSMCH"
```

The object `efficiencies.relative.temp` we created is a list, in which the two interesting elements are `par` providing the efficiency estimates for all alleles, and `convergence` indicating if the algorithm managed to converge properly (0 being the value you wish). To extract from this list the efficiency estimates, you can do:

```
efficiency.relative <- efficiencies.relative.temp$par
efficiency.relative
```

```
##  [1] 1.4900 1.6769 2.2843 1.2121 1.0300 1.2576 1.2488 1.2653 1.5323 1.3761
## [11] 1.3295 1.0624 0.5685 1.9025 0.9463 1.3702 0.7396 0.7923 0.7581 1.0338
## [21] 1.4919 1.5565 0.6572 0.6449 1.3314 0.2815 1.2987 1.2650 0.8513 0.9076
## [31] 0.9876 0.6602 0.5562 0.8516 0.5246 0.6784 0.8364 1.0554 1.7521 0.8620
## [41] 1.1692 2.2392 1.0249 0.5327 1.1127 0.7498 1.5032 1.4480 1.1397 0.4499
## [51] 1.0691 3.5717 1.1237 0.7613 0.5902 1.9996 2.0060 0.7760 1.3247 1.4434
## [61] 1.1021
```

2.4 Estimating the standardised amplification efficiency of each alleles

Efficiency estimates provided by `optim()` are relative values. An easy way to understand that is to imagine an amplicon with 4 alleles, 3 having an identical efficiency and 1 having an efficiency twice higher as the others, and then to simulate the number of reads of each allele, given that the total number of reads is 100, in two conditions: 1) with efficiences equals to 1,1,1 and 2; 2) with efficiency equals to 0.7,0.7,0.7,1.4. Simulation are performed using the function `rmultinom()` from R that we will reuse later.

```
set.seed(1)
```

```r
t(rmultinom(n = 1, size = 100, prob = c(1, 1, 1, 2)))

##      [,1] [,2] [,3] [,4]
## [1,]   17   19   22   42

set.seed(1)
t(rmultinom(n = 1, size = 100, prob = c(0.7, 0.7, 0.7, 1.4)))

##      [,1] [,2] [,3] [,4]
## [1,]   17   19   22   42
```

As you can see, both simulations provide the same number of reads (we used `set.seed()` to provide the same seed to the random generator used by `rmultinom()`, so that differences would not be just caused by randomness). This is not surprising as we said before that efficiencies are transformed into probabilities summing to 1, but it shows that `optim()` cannot estimate the absolute efficiency of alleles and depending on the optimisation and starting point for parameter values you may obtain different values of amplification efficiencies at the end of the optimization process.

We therefore use the allele Desu-DRB*1a as a reference (cf main document). This allele is called `"X001a_CP"` here. By dividing efficiency estimates by this value, we obtain standardised efficiency estimates that are no longer dependent on the starting point and the trajectory of the optimisation process. Note however that except for detecting potentially duplicated alleles, all computations from below are independent from the allele used as a reference and could be performed directly on the relative amplification efficiency values.

```r
reference <- efficiency.relative[which(colnames(matrix.data) == "X001a_CP")]
efficiency.standardised <- efficiency.relative/reference

efficiency.standardised

##  [1] 1.0000 1.1255 1.5331 0.8135 0.6913 0.8440 0.8381 0.8492 1.0284 0.9236
## [11] 0.8923 0.7130 0.3815 1.2769 0.6351 0.9196 0.4964 0.5317 0.5088 0.6938
## [21] 1.0013 1.0446 0.4410 0.4328 0.8935 0.1890 0.8716 0.8490 0.5713 0.6091
## [31] 0.6629 0.4431 0.3733 0.5715 0.3521 0.4553 0.5613 0.7083 1.1759 0.5785
## [41] 0.7847 1.5028 0.6879 0.3575 0.7468 0.5033 1.0089 0.9718 0.7649 0.3020
## [51] 0.7175 2.3971 0.7541 0.5109 0.3961 1.3420 1.3463 0.5208 0.8891 0.9687
## [61] 0.7397
```

We make a quick plot of the amplification efficiency values:

```r
plot(efficiency.standardised)
```

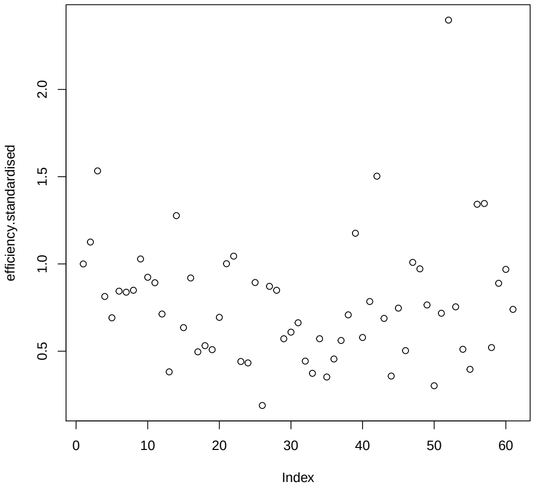

Figure 1: Fast and dirty plot of standardised amplification efficiency values

Or a tad more elaborated plot:

```r
par(mar = c(6, 6, 1, 1), mgp = c(4.5, 1, 0))
plot.new()
plot.window(ylim = c(0, 2.5), xlim = c(1, nb.alleles))
abline(h = 1)
segments(x0 = 1:nb.alleles, y0 = 1, y1 = efficiency.standardised, col = "grey")
abline(h = 2, lty = 2)
points(efficiency.standardised, col = "blue", pch = 20)
axis(2, las = 2, cex = 1.5)
name.alleles <- substring(colnames(matrix.data), first = 2)
axis(1, labels = name.alleles, at = 1:nb.alleles, las = 2, cex = 0.5)
box()
title(ylab = "Standardised efficiency", xlab = "Alleles", cex.lab = 1.5)
```

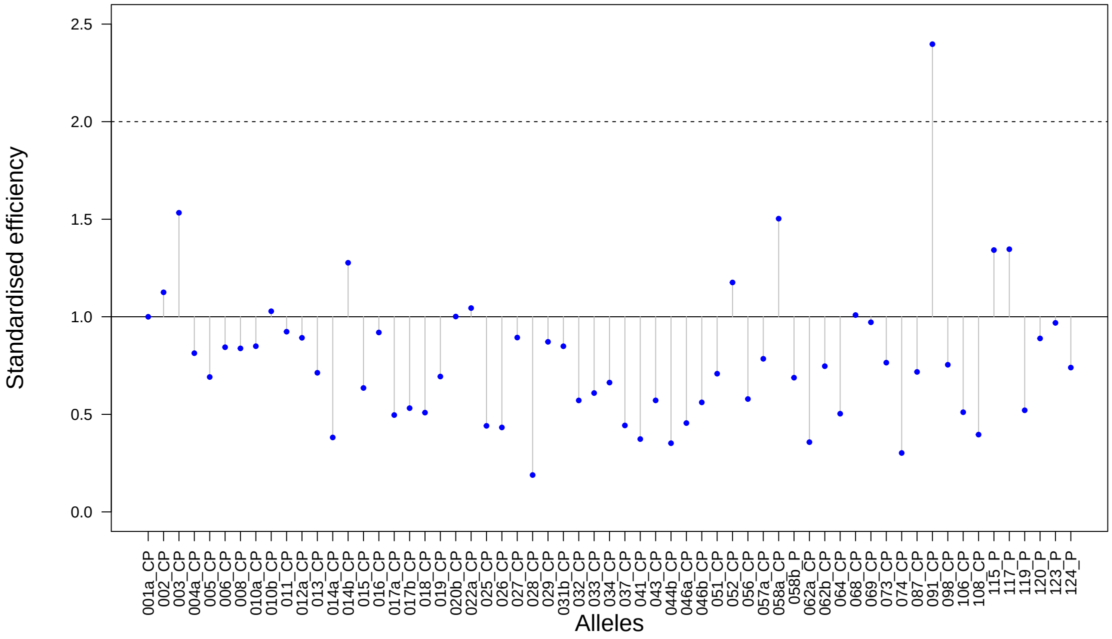

Figure 2: A better plot of standardised amplification efficiency values

3 Estimation of the minimum number of reads needed

In this section, we will describe how to compute the minimum number of reads necessary to reach a coverage of 99.9% for each genotype, using different set of assumptions.

3.1 Replicating T1 values from Galan et al. (2010)

In their excellent paper, Galan et al. (3) provide the minimum number of reads, called T1, necessary to reach a certain genotype coverage assuming that all alleles present the same efficiency. The results they present are derived from heavy computations involving the negative multinomial distribution. Here, we instead propose a much simpler and computationally lighter alternative method, which relies on using simulations involving the multinomial distribution. Note that our method leads only to an approximation of the exact results derived from the negative multinomial distribution. However, it turns out that in practice the approximation is very good and due to its fast computational performance it allows one to easily explore more realistic scenarios than those envisaged by Galan et al., which fully justifies our alternative simulation-based approach.

We first need a function that will simulate genotypes and return the coverage for a given number of reads:

```
SimuGalan <- function(nbAlleles, totalReads, nbSimulations=10000L,
```

```r
 minNbReadsPerAllele=2L) {
    # Compute the confidence of the genotyping coverage according to Galan.
    #
    # Args:
    #   nbAlleles: the number of alleles.
    #   totalReads: the total number of reads across alleles.
    #   nbSimulation: the number of simulation of read counts per genotype.
    #   minNbReadsPerAllele: the minimum number of reads for each allele,
    #     so that the genotype is considered covered.
    #
    # Returns: the proportion of simulations leading to covered genotypes.
    efficiencies <- rep(1L, nbAlleles)
    read.counts <- rmultinom(n=nbSimulations, size=totalReads, prob=efficiencies)
    is.covered <- apply(read.counts, 2, function(x) all(x >= minNbReadsPerAllele))
    frequency.of.coverage <- mean(is.covered)
    return(frequency.of.coverage)
}
```

Let's try the function to get the coverage for genotypes of 4 alleles covered by 40 reads by simulating 10,000 read counts per genotype (this is the value we set by default for the number of simulations, we recommend using at least this number):

```r
set.seed(1)
SimuGalan(nbAlleles = 4L, totalReads = 40L)

## [1] 0.9994
```

Once more, we do not need to use `set.seed()`. It is just to ensure that you can replicate the same results than here. Without setting `set.seed()`, the result becomes stochastic because the simulated genotypes will be different (just by chance).

Now, we want a function that will compute the minimum number of reads needed to reach a certain coverage (here 99.9%), i.e. T1:

```r
GalanT1Estimation <- function(nbAlleles, minReadNb=1, minCoverageConfidence=0.999,
```

```
    nbSimulations=10000L, minNbReadsPerAllele=2L, penalty=10L) {
    # Compute the minimum number of reads required to reach a certain confidence
    #   of the genotyping following Galan's assumptions.
    #
    # Args:
    #   nbAlleles: the number of alleles.
    #   minReadNb: the minimum value assumed for T1.
    #   minCoverageConfidence: the minimum acceptable confidence for genotyping
    #   nbSimulations: the number of simulation of read counts per genotype.
    #   minNbReadsPerAllele: the minimum number of reads for each allele,
    #     so that the genotype is considered covered.
    #   penalty: the correction factor to reduce minReadNb,
    #    if this latter is set too high.
    #
    # Returns: T1, the minimum number of reads required to reach a certain
    #  confidence of the genotyping.
    T1 <- max(minReadNb, 1L)
    while (T) {
        coverage <- SimuGalan(nbAlleles, T1, nbSimulations, minNbReadsPerAllele)
        if (coverage >= minCoverageConfidence) {
            if (T1 == minReadNb) {
                warning(paste("minReadNb is too high;",
                    " the function will use minReadNb = ", minReadNb-penalty))
                T1 <- GalanT1Estimation(nbAlleles, minReadNb=minReadNb-penalty,
                    minCoverageConfidence, nbSimulations, minNbReadsPerAllele,
                    penalty)
            }
            return(T1)
        }
      T1 <- T1 + 1L
    }
}
```

So if we want to estimate T1 for 4 alleles, we just need to do:

```
set.seed(1)
T1.4.alleles <- GalanT1Estimation(nbAlleles = 4L)

T1.4.alleles

## [1] 36
```

To find the minimum number of reads required (i.e. T1), the function starts by a too small T1 value (1 by default) and then increment this value untill the coverage becomes sufficiant. The argument `minReadNb` allow saving computing time by starting the incrementation of T1 to a higher value than 1. If this argument is however set to a too high value, we programmed a recursive call to the function (i.e. the function calls itself) that will use a smaller initial value for T1 (the number of units being removed is set by the argument `penalty`).

The same example as above, with a too high value for `minReadNb` as a starting point:

```
set.seed(1)
```

```
T1.4.alleles.bis <- GalanT1Estimation(nbAlleles = 4L, minReadNb = 50L)

## Warning:  minReadNb is too high;  the function will use minReadNb =  40
## Warning:  minReadNb is too high;  the function will use minReadNb =  30


T1.4.alleles.bis

## [1] 38
```

Because the result is stochastic, we might want to perform several rounds of simulations (here 100) and take the median value among the T1 values obtained. In order to save computing time, we will set `minReadNb` a bit bellow than the number we just found. We will also measure how long it takes:

```
set.seed(1)
system.time(
 T1.4.alleles.replicated <- replicate(100L, GalanT1Estimation(
      nbAlleles=4L, minReadNb=T1.4.alleles - 5L)))

##    user  system elapsed
##  38.690   1.012  41.126


median(T1.4.alleles.replicated)

## [1] 38

range(T1.4.alleles.replicated)

## [1] 36 40
```

To match our workflow, we considered that each allele has to be sequenced at least twice for a genotype to be covered but we can also easily replicate Galan's T1 assuming that 3 reads are needed as the authors did:

```
set.seed(1)
T1.4.alleles.as.Galan <- GalanT1Estimation(nbAlleles=4L, minNbReadsPerAllele=3L)
system.time(
  T1.4.alleles.as.Galan.replicated <- replicate(100L, GalanT1Estimation(
      nbAlleles=4L, minReadNb=T1.4.alleles.as.Galan - 5L, minNbReadsPerAllele=3L)))

##    user  system elapsed
##  32.470   0.772  34.345


median(T1.4.alleles.as.Galan.replicated)

## [1] 46

range(T1.4.alleles.as.Galan.replicated)

## [1] 43 48
```

3.2 Taking amplification efficiency differences into account to compute T1

To estimate the coverage confidence associated with a number of reads and taken into account efficiency variation, we can use the same simulation process as the one we used to replicate Galan's T1, meanwhile considering the amplification efficiencies we estimated (rather than an efficiency of 1 for all alleles). We will predict the coverage for the genotypes present in our dataset `matrix.data`. The function we need is very similar to the function `SimuGalan()` and will use the object `efficiency.estimates.standardised` in which standardised amplification efficiencies have been stored. Rather than the number of alleles (argument `nbAlleles` in the function `SimuGalan()`), the function `SimuVariableEfficiencies()` requires the argument `row`, which will allow the function to know which amplification efficiencies to consider to simulate read counts.

```
SimuVariableEfficiencies <- function(row, totalReads, efficiencies,
   nbSimulations=10000L, minNbReadsPerAllele=2L) {
   # Compute the confidence of the genotyping coverage taking
   #  amplification efficiencies into account.
   #
   # Args:
   #   row: the entire row of read counts that is used to identify the alleles.
   #   totalReads: the total number of reads across alleles.
   #   efficiencies: the vector of all allele efficiencies (ordered as in 'row')
   #   nbSimulations: the number of simulation of read counts per genotype.
   #   minNbReadsPerAllele: the minimum number of reads for each allele,
   #     so that the genotype is considered covered.
   #
   # Returns: the the proportion of simulations leading to covered genotypes.
   efficiencies <- efficiencies[which(row!=0L)]
   read.counts <- rmultinom(n=nbSimulations, size=totalReads, prob=efficiencies)
   is.covered <- apply(read.counts, 2, function(x) all(x >= minNbReadsPerAllele))
   frequency.of.coverage <- mean(is.covered)
   return(frequency.of.coverage)
}
```

Let's try this function by estimating the coverage obtained when the total number of reads is 40 for the 5th genotype of our dataset, which contains 4 alleles:

```
set.seed(1)
SimuVariableEfficiencies(row=matrix.data[5, ], totalReads=40L,
 efficiencies=efficiency.standardised)

## [1] 0.9365
```

Now, we want a function that will compute the minimum number of reads needed to reach a certain coverage (here 99.9%), i.e. T1, taking variation in amplification efficiencies into account:

```
VariableEfficienciesT1Estimation <- function(row, efficiencies, minReadNb=1L,
```

```
    minCoverageConfidence=0.999, nbSimulations=10000L, minNbReadsPerAllele=2L,
    penalty=10L) {
    # Compute the minimum number of reads required to reach a certain confidence
    #   of the genotyping considering variation in amplification efficiencies.
    #
    # Args:
    #   row: the entire row of read counts that is used to identify the alleles.
    #   efficiencies: the vector of all alleles efficiencies (ordered as in 'row')
    #   minReadNb: the minimum value assumed for T1.
    #   minCoverageConfidence: the minimum acceptable confidence for genotyping
    #   nbSimulations: the number of simulation of read counts per genotype.
    #   minNbReadsPerAllele: the minimum number of reads for each allele,
    #     so that the genotype is considered covered.
    #   penalty: the correction factor to reduce minReadNb,
    #     if this latter is set too high.
    #
    # Returns:
    #   T1, the minimum number of reads required to reach a certain
    #     confidence of the genotyping.
    T1 <- max(minReadNb, 1L)
    while (T) {
        coverage <- SimuVariableEfficiencies(row, T1, efficiencies,
            nbSimulations, minNbReadsPerAllele)
        if (coverage >= minCoverageConfidence) {
            if (T1 == minReadNb) {
                warning(paste("minReadNb is too high;",
                    " the function will use minReadNb = ", minReadNb-penalty))
                T1 <- VariableEfficienciesT1Estimation(row, efficiencies,
                    minReadNb=minReadNb-penalty, minCoverageConfidence,
                    nbSimulations, minNbReadsPerAllele, penalty)
            }
            return(T1)
        }
      T1 <- T1 + 1L
    }
}
```

So if we want to estimate T1 for the 5th genotype of our dataset, we just need to do:

```
set.seed(1)
T1.5th.genotype <- VariableEfficienciesT1Estimation(row=matrix.data[5, ],
    efficiencies=efficiency.standardised)

T1.5th.genotype

## [1] 80
```

Again, because the result is stochastic, we might want to perform several round of simulations (here 100) and take the median value among the T1 values obtained. Here, because variation in T1 estimation are higher between rounds of simulation, we set the argument `minReadNb` to 10 reads below our first estimation.

```
set.seed(1)
system.time(
  T1.5th.genotype.replicated <- replicate(100L, VariableEfficienciesT1Estimation(
      row=matrix.data[5, ], efficiencies=efficiency.standardised,
      minReadNb=T1.5th.genotype - 5L)))

## Warning:  minReadNb is too high;  the function will use minReadNb =  65
## Warning:  minReadNb is too high;  the function will use minReadNb =  65
## Warning:  minReadNb is too high;  the function will use minReadNb =  65
## Warning:  minReadNb is too high;  the function will use minReadNb =  65
## Warning:  minReadNb is too high;  the function will use minReadNb =  65
## Warning:  minReadNb is too high;  the function will use minReadNb =  65
## Warning:  minReadNb is too high;  the function will use minReadNb =  65
## Warning:  minReadNb is too high;  the function will use minReadNb =  65
## Warning:  minReadNb is too high;  the function will use minReadNb =  65
## Warning:  minReadNb is too high;  the function will use minReadNb =  65

##    user  system elapsed
##  23.873   0.588  25.313
```

Some warning signals are present because for some rounds of simulations `minReadNb` is too high. Still, as this does not happen very often (occurence << 100), it is still worth not reducing `minReadNb` to gain computing speed. For the few instances for which `minReadNb` is too high, the function will just perform one aditional set of simulation.

```
median(T1.5th.genotype.replicated)

## [1] 77

range(T1.5th.genotype.replicated)

## [1] 75 82
```

3.3 Estimation of the effective T1 by resampling

The previous values of T1 assume that the efficiency of an allele is independent from the genotype in which it is expressed. To relax this assumption, we can assume that the observed read frequency within a genotype are the best estimates of the amplification efficiencies of the alleles within the genotype. Calculating T1 under this consideration will also allow to assess what would have been the minimum total number of reads needed to identify the same genotypes than we did.

To do so, we first need a function that will perform the resampling:

```
ResampleReadCounts <- function(row, nbSimulations, totalReads) {
```

```
  # Resample reads counts within a genotype
  # Args:
  #   row: the entire row of read counts.
  #   nbSimulations: the number of coverage simulated.
  #   totalReads: the total number of reads across alleles after resampling.
  #
  # Returns: the simulated coverages.
  allele.indexes <- which(row!=0L)
  reads <- rep(names(row[allele.indexes]), row[allele.indexes])
  reads <- factor(reads, levels=names(row[allele.indexes]))
  read.counts <- t(replicate(nbSimulations,
      table(sample(reads, totalReads, replace=T))))
  return(read.counts)
}
```

We will try the function `ResampleReadCounts` on the 5th genotype that has the following observed number of reads:

```
row <- matrix.data[5, ]
reads <- row[which(row != 0L)]

reads

##   X006_CP X017a_CP X058a_CP X062a_CP
## 5     114       28      235       39
```

Let's perform 10 simulations of coverage by resampling as many reads as the total number of reads:

```
set.seed(1)
ResampleReadCounts(row=matrix.data[5, ], nbSimulations=10L,
    totalReads=sum(reads))

##
##         X006_CP X017a_CP X058a_CP X062a_CP
##    [1,]     112       31      232       41
##    [2,]     100       22      249       45
##    [3,]     128       29      215       44
##    [4,]     126       23      233       34
##    [5,]     130       25      220       41
##    [6,]     112       31      235       38
##    [7,]     122       31      223       40
##    [8,]     106       24      245       41
##    [9,]     111       40      219       46
##   [10,]     130       28      216       42
```

We program the function that will provide the confidence of coverage for a given genotype and total number of reads:

```
SimuResamples <- function(row, totalReads, nbSimulations=10000L,
```

```r
    minNbReadsPerAllele=2L) {
    # Compute the confidence of the genotyping coverage by resampling.
    #
    # Args:
    #   row: the entire row of read counts that is used to identify the alleles.
    #   totalReads: the total number of reads across alleles.
    #   nbSimulations: the number of simulation of read counts per genotype.
    #   minNbReadsPerAllele: the minimum number of reads for each allele,
    #     so that the genotype is considered covered.
    #
    # Returns: the the proportion of simulations leading to covered genotypes.
    read.counts <- ResampleReadCounts(row, nbSimulations, totalReads)
    is.covered <- apply(read.counts, 1, function(x) all(x >= minNbReadsPerAllele))
    frequency.of.coverage <- mean(is.covered)
    return(frequency.of.coverage)
}
```

We try the function on the 5th genotype, using first the threshold value provided by Galan's method as the number of reads (remember that the 5th genotype has 4 alleles), and then using T1 computed with the method taking efficiencies into account :

```r
set.seed(1)
T1.5th.genotype.Galan <- median(T1.4.alleles.replicated)
SimuResamples(row=matrix.data[5, ], totalReads=T1.5th.genotype.Galan)

## [1] 0.6468


T1.5th.genotype.variable.efficiencies <- median(T1.5th.genotype.replicated)
SimuResamples(row=matrix.data[5, ],
    totalReads=T1.5th.genotype.variable.efficiencies)

## [1] 0.9657
```

Now, we want a function that will compute T1 by resampling:

```r
ResampleT1Estimation <- function(row, minReadNb=1L,
```

```
    minCoverageConfidence=0.999, nbSimulations=10000L, minNbReadsPerAllele=2L,
    penalty=10L) {
    # Compute the minimum number of reads required to reach a certain confidence
    #   of the genotyping using resampling.
    #
    # Args:
    #   row: the entire row of read counts that is used to identify the alleles.
    #   minReadNb: the minimum value assumed for T1.
    #   minCoverageConfidence: the minimum acceptable confidence for genotyping
    #   nbSimulations: the number of simulation of read counts per genotype.
    #   minNbReadsPerAllele: the minimum number of reads for each allele,
    #     so that the genotype is considered covered.
    #   penalty: the correction factor to reduce minReadNb,
    #     if this latter is set too high.
    #
    # Returns:
    #   T1, the minimum number of reads required to reach a certain
    #     confidence of the genotyping.
  T1 <- max(minReadNb, 1L)
  while (T) {
        coverage <- SimuResamples(row, T1, nbSimulations,
            minNbReadsPerAllele)
        if (coverage >= minCoverageConfidence) {
            if (T1 == minReadNb) {
                warning(paste("minReadNb is too high;",
                    " the function will use minReadNb = ", minReadNb-penalty))
                T1 <- ResampleT1Estimation(row, minReadNb=minReadNb-penalty,
                    minCoverageConfidence, nbSimulations, minNbReadsPerAllele,
                    penalty)
            }
            return(T1)
        }
      T1 <- T1 + 1L
    }
}
```

Let's try the function on the 5th genotype, using `T1.5th.genotype.Efficiencies` as a starting point to save computing time:

```
set.seed(1)
system.time(
  T1.5th.genotype.resample <- ResampleT1Estimation(row=matrix.data[5, ],
     minReadNb=T1.5th.genotype.variable.efficiencies))

##    user  system elapsed
## 123.088   3.144 131.133


T1.5th.genotype.resample

## [1] 132
```

Let's now perform 100 rounds of T1 estimation as before:

```
system.time(
  T1.5th.genotype.resample.replicated <- replicate(100L, ResampleT1Estimation(
      row=matrix.data[5, ], minReadNb=T1.5th.genotype.resample - 5L)))

## Warning:  minReadNb is too high;  the function will use minReadNb =  117
## Warning:  minReadNb is too high;  the function will use minReadNb =  117
## Warning:  minReadNb is too high;  the function will use minReadNb =  117
## Warning:  minReadNb is too high;  the function will use minReadNb =  117
## Warning:  minReadNb is too high;  the function will use minReadNb =  117

##    user  system elapsed
## 1061.31   26.53 1128.66


median(T1.5th.genotype.resample.replicated)

## [1] 130

range(T1.5th.genotype.resample.replicated)

## [1] 125 135
```

3.4 Estimation of T1 for different minimum amplification efficiencies

In this section, we will describe how to compute the minimum number of reads necessary to reach a coverage of 99.9% for each genotype, when the minimum amplification efficiency is given. To do so, we can use a pair of functions very similar to the pair `SimuVariableEfficiencies - VariableEfficienciesT1Estimation`, but the new functions will take different arguments:

```
SimuMinimumEfficiency <- function(nbAlleles, minimumEfficiency, totalReads,
   nbSimulations=10000L, minNbReadsPerAllele=2L) {
   # Compute the confidence of the genotyping coverage taking
   #  minimum amplification efficiency into account.
   #
   # Args:
   #   nbAlleles: the number of alleles.
   #   minimumEfficiency: the minimum amplification efficiency considered.
   #   totalReads: the total number of reads across alleles.
   #   nbSimulations: the number of simulation of read counts per genotype.
   #   minNbReadsPerAllele: the minimum number of reads for each allele,
   #     so that the genotype is considered covered.
   #
   # Returns: the the proportion of simulations leading to covered genotypes.
   efficiencies <- c(minimumEfficiency, rep(1, nbAlleles-1))
   read.counts <- rmultinom(n=nbSimulations, size=totalReads, prob=efficiencies)
   is.covered <- apply(read.counts, 2, function(x) all(x >= minNbReadsPerAllele))
   frequency.of.coverage <- mean(is.covered)
   return(frequency.of.coverage)
}
```

Let's try this function by computing the coverage for 4 alleles, a minimum amplification efficiency of 0.7, and when the total number of reads is 36 (i.e. the T1 value under Galan's et al. assumptions):

```r
set.seed(1)
SimuMinimumEfficiency(nbAlleles=4L, minimumEfficiency=0.7, totalReads=36L)

## [1] 0.9943
```

185 We define the function getting T1:

```r
MinimumEfficiencyT1Estimation <- function(nbAlleles, minimumEfficiency, minReadNb=1L,
    minCoverageConfidence=0.999, nbSimulations=10000L, minNbReadsPerAllele=2L,
    penalty=10L) {
    # Compute the minimum number of reads required to reach a certain confidence
    #   of the genotyping considering a minimum amplification efficiency.
    #
    # Args:
    #   nbAlleles: the number of alleles.
    #   minimumEfficiency: the minimum amplification efficiency considered.
    #   minReadNb: the minimum value assumed for T1.
    #   minCoverageConfidence: the minimum acceptable confidence for genotyping
    #   nbSimulations: the number of simulation of read counts per genotype.
    #   minNbReadsPerAllele: the minimum number of reads for each allele,
    #     so that the genotype is considered covered.
    #   penalty: the correction factor to reduce minReadNb,
    #     if this latter is set too high.
    #
    # Returns:
    #   T1, the minimum number of reads required to reach a certain
    #     confidence of the genotyping.
    T1 <- max(minReadNb, 1L)
    while (T) {
        coverage <- SimuMinimumEfficiency(nbAlleles, minimumEfficiency, T1,
            nbSimulations, minNbReadsPerAllele)
        if (coverage >= minCoverageConfidence) {
            if (T1 == minReadNb) {
                warning(paste("minReadNb is too high;",
                    " the function will use minReadNb = ", minReadNb-penalty))
                T1 <- MinimumEfficiencyT1Estimation(nbAlleles, minimumEfficiency,
                    minReadNb=minReadNb-penalty, minCoverageConfidence,
                    nbSimulations, minNbReadsPerAllele, penalty)
            }
            return(T1)
        }
      T1 <- T1 + 1L
    }
}
```

186 Let's compute T1 when the minimal efficiency is 0.7 for the case of 4 alleles:

```r
set.seed(1)
T1.min.efficiency <- MinimumEfficiencyT1Estimation(nbAlleles=4L,
    minimumEfficiency=0.7)
T1.min.efficiency

## [1] 44
```

Again, we can replicate 100 times this computation and compute the median and the range of the 100 T1 values obtained:

```
set.seed(1)
system.time(
  T1.min.efficiency.replicated <- replicate(100L,
    MinimumEfficiencyT1Estimation(nbAlleles=4L, minimumEfficiency=0.7,
    minReadNb=T1.min.efficiency-5L)))

##    user  system elapsed
##  30.178   0.784  32.075


median(T1.min.efficiency.replicated)

## [1] 44

range(T1.min.efficiency.replicated)

## [1] 41 47
```

This was the result when considering that at least 2 reads per alleles are required for the coverage to be completed (default setting). For at least 3 reads per alleles, we obtain:

```
set.seed(1)
system.time(
  T1.min.efficiency.replicated <- replicate(100L,
    MinimumEfficiencyT1Estimation(nbAlleles=4L, minimumEfficiency=0.7,
    minReadNb=T1.min.efficiency-5L, minNbReadsPerAllele=3L)))

##    user  system elapsed
##  78.305   1.784  82.787


median(T1.min.efficiency.replicated)

## [1] 54

range(T1.min.efficiency.replicated)

## [1] 51 57
```

References

[1] R Core Team: *R: A Language and Environment for Statistical Computing*. R Foundation for Statistical Computing, Vienna, Austria 2012, [`http://www.R-project.org/`]. [ISBN 3-900051-07-0].

[2] Xie Y: *knitr: A general-purpose package for dynamic report generation in R* 2012, [`http://CRAN.R-project.org/package=knitr`]. [R package version 0.8].

[3] Galan M, Guivier E, Caraux G, Charbonnel N, Cosson J: **A 454 multiplex sequencing method for rapid and reliable genotyping of highly polymorphic genes in large-scale studies**. *BMC Genomics* 2010, **11**:296.
